# Supplementary material for: Narrative thinking lingers in spontaneous thought
Source: Nat Commun. 2022 Aug 6;13:4585. doi: 10.1038/s41467-022-32113-6 (PMC9357042; doi:10.1038/s41467-022-32113-6)
Supplement: Supplementary file 1 — Supplementary Information [file 41467_2022_32113_MOESM1_ESM.pdf]

Table of Contents

Supplemental Methods 2

Experiment 1: Free association, pre- and post-story 2

Experiment 2: Depth-of-processing word-list variant. 7

Experiment 3: Neutral cue variant. 8

Experiment 4: Manipulating depth of processing with coherent stories. 10

Supplementary Notes 12

(I) Length and composition of free association chains. 12

(II) Effects of scrambling on comprehension and transportation [Exp 1]. 12

(III) What does it mean to be transported when reading a word-scrambled story? [Exp 1]. 14

(IV) Follow-up analyses on lingering themes in post-story thoughts [Exp 1]. 15

(V) Cover task performance [Exp 2]. 18

(VI) Validating the levels of processing manipulation with free recall [Exp 2]. 19

(VII) Effects of deep processing on comprehension and transportation [Exp 2]. 19

(VIII) Lingering is not a result of using story-related cues in free association [Exp 3]. 22

(IX) Shallow processing of a coherent story reduces lingering [Exp 4]. 23

(X) Performance on comprehension and transportation [Exp 3 & 4]. 26

(XI) Is lingering volitional? [Exp 3 & 4]. 28

(XII) What does lingering feel like? [Exp 3 & 4]. 29

(XIII) To what extent does lingering change free association? Odds ratios [Exp 1, 2, 3 & 4]. 30

(XIV) Which aspects of transportation best predict lingering? [Exp 1, 3 & 4]. 32

(XV) Cross-story classification as evidence that lingering is story-specific [Exp 1 & 3]. 34

(XVI) Direct tests of pre- vs post-task theme similarity [Exp 1, 2, 3 & 4]. 36

Supplemental References 38

Supplemental Methods

Experiment 1: Free association, pre- and post-story

Experimental procedure. Participants were recruited via Amazon Mechanical Turk (AMT) or Prolific to participate in an online experiment. Participants were told that the experiment would consist of a series of "assorted cognitive tasks, including reading, writing and math". We intentionally used a vague description to obscure the goal of the experiment from our participants. The experiment consisted of nine sections: (I) *Math*; (II) *Pre-story free association;* (III) *Self-paced reading,* (IV) *Post-story free association;* (V) *Themes generation;* (VI) *Narrative transportation;* (VII) *Comprehension test;* (VIII) *Demographics and Strategy;* (IX) *Self-reported lingering*. Data were collected for four separate versions of the experiment (Carver, Carver-Replication, Carver-Rewrite and July; for details, see *Stimulus*). All versions were composed of the same sections, in the same order.

I. *Math*: Participants completed a series of eight addition or subtraction problems (e.g., 62 - 16, 83 + 51, etc.) and typed out their responses using their keyboard. These data were not analyzed and were only collected to help obscure the goal of the experiment.

II. *Pre-story free association*: Participants were informed that they would play a round of the "Word chain game". In this task, they were asked to type any words that came to mind for a total of 5-minutes. The task consisted of a blank white screen with a cue word in black font (e.g., WATER) and an empty field for text entry below it. The cue word remained onscreen for 2000 ms upon beginning free association and then faded away over 500 ms. Participants were instructed to type whatever words came to mind, as they came to mind, into the text entry field. The cue word acted as a starting point, to help participants begin generating their own free associations. Cue words were manually selected by the experimenter to be related to the story. Each story was associated with two cue words, one for pre-story free association and one for post, counterbalanced across participants (*Carver/Carver - Rewrite*: water, body; *July* - plane; secret). After typing each word, participants were instructed to press enter, causing the word to disappear from the text field and reappear in the cue position for 500 ms before fading away entirely. This procedure ensured that participants did not have access to the words they had previously generated. This task designed to be freeform, allowing participants to generate self-directed sequences of free associations for 5-minutes without interruption. The only additional direction we provided was asking participants to avoid stringing their words together to form sentences.

III. *Self-paced reading*: Participants were presented with the text, one sentence at a time. Participants progressed through the text at their own pace by pressing space bar after reading each sentence. Participants could not return to previously read sentences and could only move forward. All stories were between 2,158 to 2,798 words in length, ranging between 196 to 268 sentences. For more details regarding the stories we used, see *Stimuli*.

IV. *Post-story free association*: Post-story free association began after self-paced reading and was identical to pre-story free association, save for the use of a different cue word to begin the task. Post-story free association only began after participants finished reading a reminder of the instructions for the task, which was self-paced. Across all experiments,

participants began post-story free association an average of 26.08 seconds (SD = 13.51; min = 10.64, max = 85.38) after completing the self-paced reading section.

V. *Theme generation: After post-story free association,* participants completed a series of self-report questionnaires and comprehension questions. First, participants were asked to “pick 10 words that relate to the central themes and ideas” of the text they read. These words acted as anchors for the word embedding analyses described in the manuscript. Participants who could not come up with 10 words came up with as many as they could before proceeding to the next section of the experiment.

VI. *Narrative Transportation*: Next, participants completed an edited version of the Narrative Transportation Questionnaire [1]. The Narrative Transportation Questionnaire was composed of 13 items assessing the extent to which participants were transported into the story while reading it (e.g., “While I was reading the text, I could easily picture the events in it taking place”; “I could picture myself in the scene of the events described in the text”; “The text affected me emotionally”; for all items see Supplemental Information). Participants responded to each item on a 7-point scale ranging from Not At All – 1 – to Very Much – 7, with two reverse coded items (e.g., “While reading the text, activity going on in the room around me was on my mind”; “I found my mind wandering while reading the text”). Scores for all items were summed and reported as proportions where 1 is the highest achievable score of transportation. One item was dropped from the overall transportation score (“After the text ended, I found it easy to put out of my mind”), leaving a total of 12-items and a maximum score of 84 (7 x 12 = 84) and a minimum score of 12 (1 x 12 = 12). We dropped this item to better distinguish the construct of transportation (i.e., immersion in the story *while reading it*) from lingering (i.e., the *lasting* influences the story on thought). After the Narrative Transportation Questionnaire, participants in the Carver-Replication condition additionally performed the Story World Absorption Scale (SWAS) [2]. SWAS is an 18-item questionnaire that also taps into a participant’s subjective experience of being immersed in a story.

VII. *Comprehension test*: Comprehension of verifiable story details was measured using a 26-item 4-alternative-forced-choice test. Questions were presented out of chronological order. 2 items were catch trials and the remaining 24 assessed comprehension of the intact story. Half of the content questions were very general (e.g., “Which of the following beverages figured most prominently in the passage?”) while the remaining half were specific and plot-focused (e.g., “How did Claire’s husband encounter the body?”). Questions for both stories can be found in online (https://osf.io/dmbx4/).

VIII. *Demographics and Strategy*: Next, participants completed a series of demographics and strategy-related questions. Demographics questions measured age, gender, handedness, language proficiency, race and ethnicity, education, reading habits, and whether or not participants had read the story from the experiment before. In terms of strategies, participants were asked to describe any strategies they may have used while performing the Math section or the Free association sections.

IX. *Self-reported lingering*: Next, participants were asked about their subjective experience of the text “lingering” in their minds. Specifically, participants were asked to (i) describe any differences they felt between pre- and post-story free association and (ii) provide a rating of their experience of the text lingering in their minds (i.e., “To what extent did the text linger in your mind after reading it?”) on a scale of 1 (Not At All) to 7 (Very Much).

These questions were included at the end of the experiment to further help obscure the purpose of the experiment from our participants.

Stimuli. During the self-paced reading task, participants were presented with one of 3 stories:

1. *So Much Water So Close To Home* by Raymond Carver (*Carver; Carver-Replication*) [3]
2. A rewrite of Carver, conveying the same story using different words (*Carver-Rewrite*);
3. *Roy Spivey* by Miranda July (*July*) [4].

These stories were chosen because they were easy to read (at a Grade 5 reading level, or below), short (under 3000 words), yet still immersive and evocative. Carver was 2,357 words in length, broken into 268 sentences, and was highly readable with a Flesch-Kincaid reading grade level of 2.26. Carver - Rewrite was 2,158 words in length, broken into 196 sentences, with a higher Flesch-Kincaid reading grade level of 5.04. July was 2,798 words in length, broken into 265 sentences, with a Flesch-Kincaid reading grade level of 3.79.

These three stories also varied to different degrees in terms of plot, themes, and valence. This was intentional, as it allowed us to ensure the lingering effects reported here were generalizable across stories. Participants in the Carver and Carver-Replication versions of the experiment read Raymond Carver's *So Much Water So Close to Home*. It is about the relationship between a husband, Stuart, and wife, Claire. The story is told from Claire's perspective and centers around a camping trip, where the husband and his friends found the body of a dead girl. Upon returning home, Claire begins to consider the possibility that Stuart may have committed the murder and the story concerns itself with Claire coming to terms with this realization and its consequences for her relationship with Stuart. Participants in the Carver-Rewrite condition read a paraphrased version of Raymond Carver's *So Much Water So Close to Home*, re-written by an experimenter (BB). The story has the same plot and themes as the original, while using different words. The rewrite was also written in 3rd person, rather than first, and uses longer sentences (note the relatively higher Flesch-Kincaid reading grade level) and less direct dialogue between characters. Participants in the July version of the experiment read *Roy Spivey* by Miranda July. *Roy Spivey* is a more positive and upbeat story about an unnamed protagonist who was coincidentally seated beside a famous Hollywood actor on a flight. The story is told from the protagonist's point of view and captures the humorous, flirty and sincere nature of their chance encounter. After the plane lands, the actor gives the protagonist his phone number, withholding one number – the number four – which he asks her to commit to memory. With this secret phone number in hand, the protagonist is faced with a decision about whether or not she will return to her normal life.

Participants and exclusion criteria. 1012 participants took part in Experiment 1 and were recruited via Amazon Mechanical Turk (versions: Carver, Carver-Rewrite, July) or Prolific (version: Carver-Replication). MTurk data were collected over the span of June 2019 – March 2020. Prolific data were collected during September 2020. The experiment lasted approximately 45 minutes. Participants were paid $6.00 USD for their participation and provided informed consent before participating. The study protocol was approved by the Johns Hopkins University Homewood Institutional Review Board.

In line with recently published recommendations for online testing, we included a variety of data quality assurance indicators [5]. Participants were excluded from the final sample if they failed to meet *any* of the following criteria:

(i) *Useable free association data*: In free association, participants were required to type words for 5-minutes, before and after reading the story. Participants were excluded from the final sample if their pre- or post-story free association chains consisted of: 20 or fewer words; complete sentences rather than individual words (e.g., “the”, “reason”, “to”, “have”, “a”, “medium”…); one third or more of the same word (e.g., “body”, “body”, “body”, “body”…); or a majority of gibberish or nonwords (e.g., “xcv”, “xcv”, “xcv”, “cvxxcv”, “xcv”…).

(ii) *Above chance comprehension*: For the intact and sentence-scrambled conditions, we could expect participants to show above chance comprehension for the verifiable details of the story they read. Therefore, participants who performed at or below chance (25%) on the general multiple-choice questions were excluded from the final sample. Note that this criterion was not used for the participants in the word-scrambled condition.

(iii) *Remaining on-task*: Anytime a participant clicked away from the browser window in which the experiment was presented, it was recorded as an “off-task” event. Participants who were outliers in terms of off-task events were excluded from the experiment. An outlier was defined as the third quartile (Q_3) + 2.5 * interquartile range (IQR), which was calculated separately per dataset (i.e., Carver, Carver-Replication, Carver-Rewrite, July).

(iv) *Missed catch trial*: Two catch trials were included in the multiple-choice comprehension test to ensure participants were paying attention. One of the catch trials was dropped as many participants found it confusing. Participants who responded incorrectly to the remaining catch trial (i.e., “In what language was the passage written?”) were dropped.

(v) *No breaks between reading and post-story free association*: Overall, the experiment was broken up into sections, each beginning with instructions that participants read at their own pace. Because of this, some participants appeared to take extended breaks between the self-paced reading and post-story free association sections. To this end, participants who were outliers in the duration between the end of self-paced reading and the beginning of post-story free association were dropped. Again, an outlier was defined as the Q_3 + 2.5 * IQR, which was calculated separately per dataset.

As mentioned in (ii), participants in the word-scrambled condition were not required to show above chance performance on the general comprehension questions. Instead, participants in this condition were required to *either* show 80% accuracy on the probe detection task (for details, see Story-scrambling manipulation) or a statistically significant correlation ($p < 0.05$) between sentence-level reading times in the self-paced reading task and the number of characters in a sentence. If a participant in the word-scrambled condition failed to meet this criterion or (i), (iii), (iv) and (v) above, they were excluded from the final sample.

After exclusions, a total of 720 participants were included in the final sample (N_{male} = 360; N_{female} = 354, with 6 participants selecting “None of the above / Prefer not to identify”). Eighty participants were included in each condition, per story: Carver [Intact/Sentence-scrambled/Word-scrambled], Carver-Replication [Intact/Sentence-scrambled], Carver-Rewrite [Intact/Sentence-scrambled], and July [Intact/Sentence-scrambled]. Median age range in the final sample was 35-39 years of age (Q_2 = 25-29, Q_3 = 45-49, min = 18-19, max = 70-74).

Median level of completed education was a bachelor's degree (Q_2 = "Some college but no degree", Q_3 = "Bachelor's degree", min = "Less than high school", max = "Doctoral degree"). The majority of our participants identified as White (80%), followed by African American/Black (6.80%), Asian (6.25%), Multiracial (4.31%), Indigenous/Native American (0.55%), and 2.08% choosing not to identify. We additionally asked our participants how often they read for pleasure. Median reported frequency of reading for pleasure was once a week (Q_2 = "More once a year, but less than once a month", Q_3 = "More than once a week, but less than once a day", min = "Never", max = "More than once a day").

Analysis of free association chains.

Part-of-speech tagging. Part-of-speech tagging was implemented using the RDRPOSTagger package in R [6]. Each free association chain was parsed into its constituent parts-of-speech and overall means and standard deviations are reported.

Document classification. Document classification was implemented in R. Document term matrices were calculated using the tm package [7], with sparsity set at 0.99. A sparsity level of 0.99 implies that a word must occur in at least 2 documents to be included in the document term matrix. Support vector machine classifiers were implemented using *svm()* from the e1071 package [8], using a linear kernel.

Word-embeddings and "theme similarity". Document classification provided a data-driven test of whether free association chains were discriminable pre- vs. post-story. However, in order to directly test the hypothesis that *story themes* were driving the post-story lingering effects, we turned to word-embeddings. Word embedding techniques provide a low-dimensional vector mapping of word meaning. Specifically, each word in a corpus occupies a position in an n-dimensional space, where words occupying similar positions in this space tend to have common features (e.g., semantic meaning). We used a freely-available, pretrained version of the Global Vectors word embeddings (GloVe; version: Wikipedia 2014 + Gigaword 5) [9]. According to the Distributional Hypothesis, words that occur in the same contexts will often have similar meanings [10]. As such, GloVe vectors, like all unsupervised methods of word representation learning, make use of the co-occurrence statistics of words in large corpora of natural language (e.g., Wikipedia) to approximate the semantic relationships between words. The GloVe algorithm was trained on the Wikipedia 2014 and Gigaword 5 (an archive of English news articles from a variety of international sources) dumps, which was composed of 6 billion tokens, of which 400 thousand were unique, amounting in 300-dimensional vectors for each unique word in the corpus. Critically, semantic similarity between words could be assessed using cosine similarity; semantically similar words tend to have corresponding vectors with high cosine similarity.

The majority of words in our participants' free association chains were then linked to a 300-dimensional vector. Free associates without corresponding vectors in pretrained corpus were dropped from subsequent analyses. To estimate the lasting influence of story themes on post-story free association, we also needed an estimate of the story themes themselves. To this end, we used the core themes reported by the participants in our experiment. Immediately following post-story free association, participants were instructed to enter 10 words that relate to the text's central themes and ideas. For each story, we then selected the 10 words that were mentioned most frequently across participants, collapsing across conditions. These words are referred to as "theme words". When collapsing across all participants, theme words for each story are as follows, from most to least frequent: Carver ["murder", "death", "funeral", "fishing", "girl", "family", "camping", "river", "beer", "sex"], Carver-Rewrite ["murder", "funeral", "wife", "husband", "death",

"fishing", "camping", "suspicion", "mystery", "friends"], and July ["four", "celebrity", "plane", "airplane", "husband", "secret", "number", "affair", "actor", "famous"].

Using the 10-theme words, we were able to directly quantify the extent to participants' free association chains were semantically similar to the story themes, before and after reading. To this end, we calculated a measure we refer to as "theme similarity". Theme similarity was calculated for each word in a free association chain using the following formula:

$$\text{theme similarity}(A_n) = \max_i \left(\frac{A_n \cdot B_i}{\| A_n \| \cdot \| B_i \|} \right)$$

where A_n is the embedding vector for a target word in a given free association chain and B_i is the embedding vector for the ith theme word. To reiterate, theme similarity is the maximum cosine similarity between a target free associate and the story's 10 theme words. By taking the maximum theme similarity, rather than a measure of central tendency (e.g., mean), the measure is sensitive to a free associate's proximity to any of the story themes and does not require any assumptions about the relative positions of theme words in the GloVe embedding space. Theme similarity was then calculated for every word in each free association chain and then averaged per chain, amounting in two measures per participant (semantic similarity to theme words pre-story and post-story), allowing for a within-participant test of how theme similarity changes from pre- to post-story. Note that the 10 theme words used for a given participant's theme similarity calculation were selected by taking the most frequent theme words produced across participants, after excluding that participant's own theme words. This leave-one-participant out procedure was included to remove any circularity from the analysis, however, for the majority of participants, the theme words calculated this way were identical if not highly comparable to those calculated from the full sample.

In order to estimate the duration of the theme similarity effects, theme similarity estimates were also depicted in 10-word windows (Figure 5B). In this analysis, average participant-specific post-story and pre-story theme similarity estimates were compared per 10-associate window using Cohen's d: $(M_{post} – M_{pre})/SD_{pooled}$. Ninety-five percent confidence intervals were estimated using in R using the *cohen.d()* function in the effsize package [11]. All participants contributed to the first two windows, as participants with fewer than 20 free associates were excluded from all analyses (see Participants and Exclusion Criteria). Data are plotted up until the sixtieth associate, as fewer than 50% of participants reliably contribute to each bin after this point.

Additional control analyses are reported in the Supplemental Results, including replicating the theme similarity effects after calculating the top 10 theme words separately per condition, and eliminating the lasting theme similarity effect when using the theme words from an unrelated story (e.g., July themes on Carver data).

Experiment 2: Depth-of-processing word-list variant.

Experimental procedure. Participants were recruited via Amazon Mechanical Turk (AMT) to participate in an online experiment. Procedures were identical to Experiment 1, except for the fact that the self-paced reading phase was replaced by incidental list-learning and Experiment 2 included additional post-story components: story description and a test of free recall (for details, see Manuscript).

Stimulus. For additional details regarding the stimulus, see Manuscript. The discriminability of words that were manually labelled as tangible vs. intangible or decoy vs. story/theme were further validated using normative ratings of concreteness [12] and valence [13], respectively. Concreteness ratings ranged between 1 (abstract) and 5 (concrete). Valence ratings ranged between 1 (unhappy) and 9 (happy). Overall, the word list was composed of words with a median concreteness of 2.6 of 5 (Q_2 = 2.1, Q_3 = 4.0, min = 1.2, max = 5.0; missing values = 22) and a median valence of 6.5 of 9 (Q_2 = 3.9, Q_3 = 7.4, min = 1.5, max = 8.5; missing values = 34). A Welch's t-test indicated that words labelled as tangible were indeed more concrete than intangible words [$M_{tangible}$ = 4.5, $M_{intangible}$ = 2.6; $t(115.14) = 20.9$, $p < 0.0001$, $d = 2.62$]. Similarly, decoy words were more positively valenced than story words [M_{decoy} = 7.7, M_{story} = 4.9; $t(219) =$ 17.0, $p < 0.0001$, $d = 1.68$].

Participants and exclusion criteria. 769 participants took part in Experiment 2 and were recruited via Amazon Mechanical Turk. Data were collected during July 2020. Participants were paid $6.00 USD for their participation and provided informed consent before participating. The study protocol was approved by the Johns Hopkins University Homewood Institutional Review Board.

Again, in line with recently published recommendations for online testing, we included a variety of data quality assurance indicators [5]. A participant was excluded from the final sample if they failed to meet *any* of the criteria from Experiment 1, with the exception of above chance performance on the comprehension test. In addition to original exclusion criteria, participants were excluded from the experiment if their summary of the hidden story was incomprehensible or obviously unrelated to the story content (e.g., "ralistic story and happiness story amezed real story for entertainted story"; "a lion and mouse. one day lion was sleeping. the mouse started playing on it.the lion wake up."; "Very useful and intereting in this survey..........very honest. this survey in very interesting and amazing").

After exclusions, a total of 320 participants were included in the final sample (N_{male} = 201; N_{female} = 113, with 6 participants selecting "None of the above / Prefer not to identify"). Eighty participants were included in each condition: Italic, Tangible, Theme and Story. Median age range in the final sample was 35-39 years of age (Q_2 = 25-29, Q_3 = 45-49, min = 18-19, max = 70-74). Median level of completed education was a bachelor's degree (Q_2 = "Some college but no degree", Q_3 = "Bachelor's degree", min = "Less than high school", max = "Doctoral degree"). The majority of our participants identified as White (74.4%), followed by African American/Black (12.50%), Asian (6.25%), Multiracial (2.50%), Indigenous/Native American (2.19%), and 2.19% choosing not to identify. Median reported frequency of reading for pleasure was once a week (Q_2 = "More once a year, but less than once a month", Q_3 = "More than once a week, but less than once a day", min = "Never", max = "More than once a day").

Experiment 3: Neutral cue variant.

Experimental procedure. Participants were recruited via Prolific to participate in an online experiment. Procedures were largely identical to those in Experiment 1, except that the pre- and post-story free association used a neutral rather than story-related cue: "Enter a word to begin!".

In addition to the neutral cue, participants in Experiment 3 performed additional self-report questionnaires, appended to the end of the procedure in Experiment 1. These questionnaires are described below:

IX. *Self-reported lingering*: After the Demographics and Strategies section (see, Experiment 1, Experimental procedure: VIII), participants were asked about their subjective experience of the text "lingering" in their minds. Specifically, participants were asked to (i) describe any differences they felt between pre- and post-story free association and (ii) provide a rating of their experience of the text lingering in their minds (i.e., "To what extent did the text linger in your mind after reading it?") on a scale of 1 (Not At All) to 7 (Very Much). These questions were included at the end of the experiment to further help obscure the purpose of the experiment from our participants.

Participants who indicated the presence of at least some lingering (i.e., a score of 2 or more on the 7-point Likert scale) were probed with additional questions on the subjective quality of lingering. Participants who reported no lingering (i.e., a score of 1) moved on to the next section.

First, participants were asked to describe the volitional nature of their experience of lingering. Specifically, participants were asked: "Were you *intentionally* reflecting on the text while playing the word chain game? Or, did aspects of text come to mind *unintentionally*?" Participants had to choose one of five potential responses: (1) "I was intentionally reflecting on the text I read", (2) "The text I read came to mind unintentionally", (3) "Both", (4) "Neither", (5) "Don't know". Next, participants were asked to elaborate on their response in a few sentences using an open-ended text response.

Next, participants were asked to describe what was lingering in their minds. To this end, participants were asked to rate the six following statements using a 7-pt Likert scale [1 (Not At All) to 7 (Very Much)]: After reading the story, I noticed… (1) "A change in the topics that came to mind while playing the word chain game"; (2) "A change in how easy or difficult it was to come up with words while playing the word chain game", (3) "A change in the emotions I felt while playing the word chain game"; (4) "A change in how tired I felt while playing the word chain game"; (5) "A change in how bored I felt while playing the word chain game"; and (6) There was a change in my thoughts, but it was not something that is captured by the questions above. Participants were again asked to elaborate on their response in a few sentences using an open-ended text response.

X. *Pre-clinical measures of depression and rumination*: After self-reported lingering, participants completed two additional scales: Depression, Anxiety and Stress Scale (DASS-21) [14]; and Rumination Response Scale (RRS-12) [15]. Data for these scales are not further discussed in this manuscript.

Stimulus. During the self-paced reading task, participants read the intact version of *So Much Water So Close To Home* by Raymond Carver.

Participants and exclusion criteria. 101 participants took part in Experiment 3 and were recruited via Prolific. Data were collected during January 2022. Participants were paid $6.92 USD for their participation and provided informed consent before participating. The study protocol was approved by the Johns Hopkins University Homewood Institutional Review Board.

After applying identical exclusion criteria from Experiment 1, a total of 80 participants were included in the final sample (N_{male} = 17; N_{female} = 63). Median age range in the final sample was 30-34 years of age (Q_2 = 20-24, Q_3 = 40-44, min = 18-19, max = 60-64). Median level of completed education was a bachelor's degree (Q_2 = "High school degree or equivalent", Q_3 = "Bachelor's degree", min = "Less than high school", max = "Doctoral degree"). The majority of

our participants identified as White (72.5%), followed by African American/Black (13.75%), Multiracial (6.25%), Asian (2.5%), and 5% choosing not to identify. Median reported frequency of reading for pleasure was once a week (Q_2 = "More than once a year, but less than once a month", Q_3 = "Once a day", min = "Almost never", max = "More than once a day").

Experiment 4: Manipulating depth of processing with coherent stories.

Experimental procedure. Participants were recruited via Prolific to participate in an online experiment. Procedures were largely identical to those in Experiment 1, except that the self-paced reading task was modified such that each sentence was presented in the context of one of two cover tasks, manipulating the depth with which participants read an intact story.

All participants were presented with an intact version of the Carver story. Participants were randomly assigned to one of two conditions, one that encouraged shallow processing of the story (i.e., Proofread) and another that encouraged deep processing (i.e., Emotion). In the Proofread condition, participants were instructed to read each sentence and indicate, through key press, the total number of errors they found. There were three response options: 0, 1, or 2. Errors consisted of words that were manually modified to either have a spelling error or a font error (i.e., font did not match the font used for the majority of words in the story). For details regarding these typographical errors, see Stimulus section below. The total number of errors was used as the response variable, because this required participants to look at the font and also to read the words. As in Experiment 2, participants received feedback after each trial in the form of a checkmark or x. In the Emotion condition, participants were instructed to indicate how each sentence contributes to the valence of that moment in the story. There were three response options: positive, negative, neutral. The participants in the Emotion condition were explicitly instructed to expect and ignore spelling and font errors in the story. No feedback was provided.

As participants read a version of the intact Carver story, identical story-related cue words from Experiment 1 were used in pre- and post-story free association.

Participants in Experiment 4 performed the same additional self-report questionnaires described above in Experiment 3.

Stimulus. During the self-paced reading task, participants read an intact version of *So Much Water So Close To Home* by Raymond Carver. The text was manually edited such that 50% of the sentences contained an "error", in which 25% contained 1 error and the remaining 25% contained two errors. Errors were either (i) in spelling (e.g., "teh" instead of "the"; or "dihses" instead of "dishes") or (ii) in font (i.e., most of the story was displayed in a sans-serif font, Arial, while the "error" words would be displayed in a serif font, Times). To ensure overall readability, only 10% of the sentences contained spelling errors. The proportion of trials for each trial type are as follows: 0 font error and 0 spelling errors (50%), 1 font error and 0 spelling errors (20.5%), 0 font errors and 1 spelling error (4.5%), 1 font error and 1 spelling error (4.5%), 2 font errors and 0 spelling errors (19.5%), 0 font errors and 2 spelling errors (1%). The values above are based on a total of 259 trials (i.e., sentences). Note that the original Carver story used in Experiment 1 had a total of 269 sentences, but 10 consisted of paragraph breaks, demarcated with a "***".

Participants and exclusion criteria. 224 participants took part in Experiment 4 and were recruited via Prolific. Data were collected during January 2022. Participants were paid $6.92

USD for their participation and provided informed consent before participating. The study protocol was approved by the Johns Hopkins University Homewood Institutional Review Board.

Experiment 4 used the same exclusion criteria as Experiment 1 with one additional criterion: participants in the Proofread condition had to reach a minimum level of accuracy for detecting errors. Specifically, we calculate *d'* [i.e., *d'* = *z*(Hit rate) – *z*(False alarm rate)] for the trials with errors, and participants were only included in the final sample if their d' was greater than or equal to 0. Note that this criterion was accidentally omitted from the original preregistration.

After applying these exclusion criteria from, a total of 160 participants were included in the final sample (N_{male} = 76; N_{female} = 80, with 4 participants selecting "None of the above / Prefer not to identify"). Eighty participants were included in each condition: Emotion or Proofread. Median age range in the final sample was 35-39 years of age (Q_2 = 25-29, Q_3 = 45-50, min = 18-19, max = 70-74). Median level of completed education was a bachelor's degree (Q_2 = "Some college but no degree", Q_3 = "Bachelor's degree", min = "Less than high school", max = "Doctoral degree"). The majority of our participants identified as White (81.88%), followed by African American/Black (8.75%), Multiracial (2.5%), Asian (1.88%), and 5% choosing not to identify. Median reported frequency of reading for pleasure was once a week (Q_2 = "More than once a year, but less than once a month", Q_3 = "More than once a week, but less than once a day", min = "Never", max = "More than once a day").

Supplementary Notes

(I) Length and composition of free association chains.

Across all datasets in Experiment 1, participants generated free association chains with an average length of 70.96 words (SD = 25.76, max = 162, min = 20) over the course of 5-minutes. Free association chains were primarily composed of nouns (M = 65.6%, SD = 11.8%), verbs (M = 14.3%, SD = 7.2%) and adjectives (M = 13.3%, SD = 8.2%).

Experiment 2: Participants generated free association chains of 65.67 words on average (*SD* = 25.75, *max* = 144, *min* = 20). Free association chains also had a similar composition to those from Experiment 1, primarily consisting of nouns (*M* = 67.4%, *SD* = 12.2%), verbs (*M* = 13.3%, *SD* = 7.6%) and adjectives (*M* = 13.2%, *SD* = 7.9%).

Experiment 3: Participants generated free association chains of 73.3 words on average (*SD* = 23.71, *max* = 147, *min* = 24). Free association chains also had a similar composition to those from Experiment 1, primarily consisting of nouns (*M* = 63.6%, *SD* = 10.3%), adjectives (*M* = 14.5%, *SD* = 7.3%) and verbs (*M* = 13.9%, *SD* = 6.8%).

Experiment 4: Participants generated free association chains of 69.29 words on average (*SD* = 23.48, *max* = 151, *min* = 20). Free association chains also had a similar composition to those from Experiment 1, primarily consisting of nouns (*M* = 66.6%, *SD* = 11.1%), adjectives (*M* = 13.9%, *SD* = 8.5%) and verbs (*M* = 12.7%, *SD* = 6.7%).

(II) Effects of scrambling on comprehension and transportation [Exp 1].

Here we report the consequences of sentence-scrambling on comprehension of verifiable story details and self-reported transportation using the data collected for our three follow-up stories (i.e., Carver-Replication, Carver-Rewrite, July-Original). Overall, comprehension and transportation were higher for the Intact as compared to the Sentence-scrambled condition for all stories. The details of the analyses are reported below (see Figures S1 and S2):

Comprehension of verifiable story details was operationalized as the proportion of correct responses on a 24-item multiple choice test. A 2 (Condition: Intact, Sentence-scrambled) x 3 (Story: Carver-Replication, Carver-Rewrite, July-Original) between-subjects ANOVA on comprehension test performance produced significant main effects of Condition [$F(1,474) = 202.89$, $p < 0.0001$, $\eta^2_G = 0.30$], Story [$F(2,474) = 30.15$, $p < 0.0001$, $\eta^2_G = 0.11$], and a significant Story x Condition interaction [$F(2,474) = 6.08$, $p = 0.002$, $\eta^2_G = 0.03$]. Paired t-tests indicated that the Intact condition was associated with better comprehension than Sentence-scrambled across all stories, though the effect size was largest for Carver-Replication [Caver-Replication: $t(158) = 9.78$, $p < 0.0001$, $d = 1.55$; Carver-Rewrite: $t(158) = 6.89$, $p < 0.0001$, $d = 1.09$; July-Original: $t(158) = 7.78$, $p < 0.0001$, $d = 1.23$).

Narrative transportation was operationalized using a modified version of the Narrative Transportation Questionnaire (Green & Brock, 2000), including 12 items, each receiving a score of 1-7. Transportation was summarized as a proportion of the maximum attainable score (i.e., 7*12 = 84). A 2 (Condition: Intact, Sentence-scrambled) x 3 (Story: Carver-Replication, Carver-Rewrite, July-Original) between-subjects ANOVA on transportation produced significant main effects of Condition [$F(1,474) = 124.13$, $p < 0.0001$, $\eta^2_G = 0.21$], Story [$F(2,474) = 11.85$, $p < 0.0001$, $\eta^2_G = 0.05$], and a significant Story x Condition interaction [$F(2,474) = 3.47$, $p = 0.03$, $\eta^2_G = 0.01$]. Paired t-tests indicated that the Intact condition was associated with higher

transportation than Sentence-scrambled across all stories, though the effect size was notably lower for Carver-Rewrite [Caver-Replication: $t(158) = 7.25$, $p < 0.0001$, $d = 1.15$; Carver-Rewrite: $t(158) = 4.63$, $p < 0.0001$, $d = 0.73$; July-Original: $t(158) = 7.32$, $p < 0.0001$, $d = 1.16$).

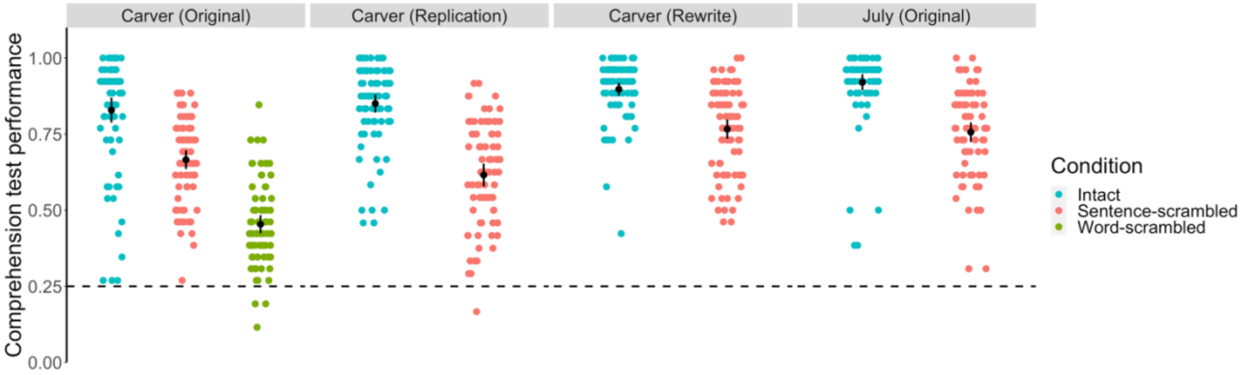

Figure S1. *Coherent stories promote memory for verifiable story details*. Distribution of comprehension test scores for all participants, across all stories and conditions in Experiment 1. Overall, reading an Intact story was associated with better comprehension than when the story was scrambled at the sentence or word-level. Comprehension of verifiable story details was operationalized as the proportion of correct responses on a 24-item multiple choice test about the content of the stories. All Carver datasets used an identical test. Each point represents a participant. Black points represent condition means. Error bars reflect 95% confidence intervals. Dashed horizontal line represents chance performance (each question had four response options, making chance = 25%). n = 80 participants per condition, per experiment. Source data are provided as a Source Data file.

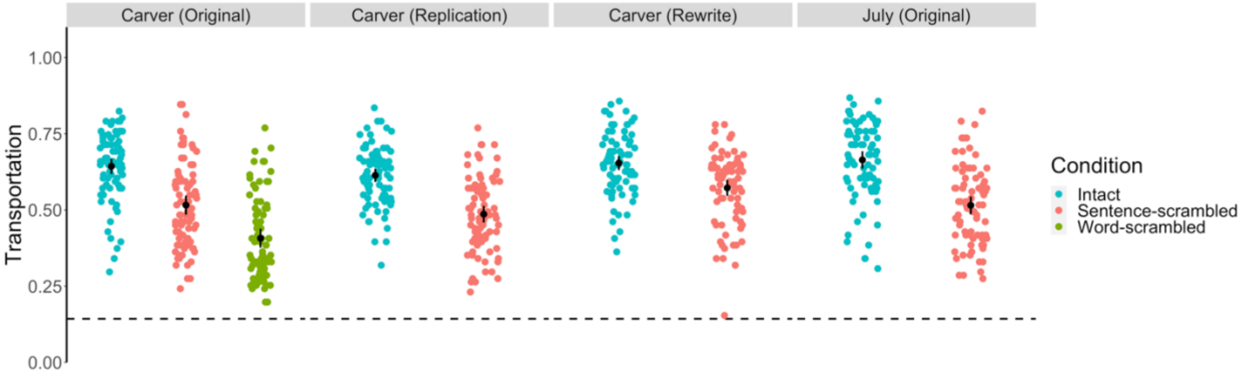

Figure S2. *Coherent stories are more likely to transport us into the world of the story*. Distribution of transportation scores for all participants, across all stories and conditions in Experiment 1. Overall, reading an Intact story was associated with stronger transportation than when the story was scrambled at the sentence or word-level. Transportation was assessed using a modified version of the Narrative Transportation Scale (Green & Brock, 2000) and a proportion of the maximum attainable score (max = 7*12 = 84). Each point represents a participant. Black points represent condition means. Error bars reflect 95% confidence intervals. Dashed horizontal line represents the lowest attainable score (each question received a rating of 1 to 7; lowest attainable score = 12/84 = 0.14). n = 80 participants per condition, per experiment. Source data are provided as a Source Data file.

(III) What does it mean to be transported when reading a word-scrambled story? [Exp 1].

To address this question, we plotted the scores for each item in the narrative transportation scale (Green & Brock, 2000) separately, particularly for those in the Carver (Original) dataset (Figure S3).

Unsurprisingly, several items showed a marked decrease for the Word-scrambled condition (e.g., Q1, Q3, Q6, Q7, Q8, Q12 and Q13; see Figure S3C). However, three items are worth noting: Q4, Q5 and Q9.

Across all conditions, participants tended to score fairly highly on Q4 (whether they “felt “mentally involved in the text while reading it”). Therefore, this aspect of the narrative transportation construct is experienced to a similar extent whether the narrative being read is intact or has its word-order randomly scrambled. This measure of domain-general involvement, perhaps reflecting the degree to which the participant was “on task”, is one of the main drivers behind why participants in the Word-scrambled condition reported feeling transported.

Q5 is highest for participants in the Word-scrambled condition. While at first this may seem odd, Q5 was a reversed scored item that, in many ways, can be considered the opposite of our lingering construct (“After reading the text, I found it easy to put out of my mind”). Therefore, a higher score on this item indicates that participants who were in the Word-scrambled condition found it easier to disengage from the text after reading. Note: this item was excluded from our all reported calculations of transportation to better distinguish between transportation (i.e., depth of engagement while reading) and lingering (i.e., persistence of an experience in mind after it ends).

Q9 was also highest for participants in the Word-scrambled condition but, again, it reflects a reverse-scored item in the transportation survey (i.e., “I found my mind wandering while reading the text”).

For a more detailed treatment of how individual items in the transportation scale predict self-reported lingering, see *SI: Supplemental Results XIV.* Also, it is worth noting that the word-order was not randomly scrambled, but instead scrambled in windows of 5-sentences.

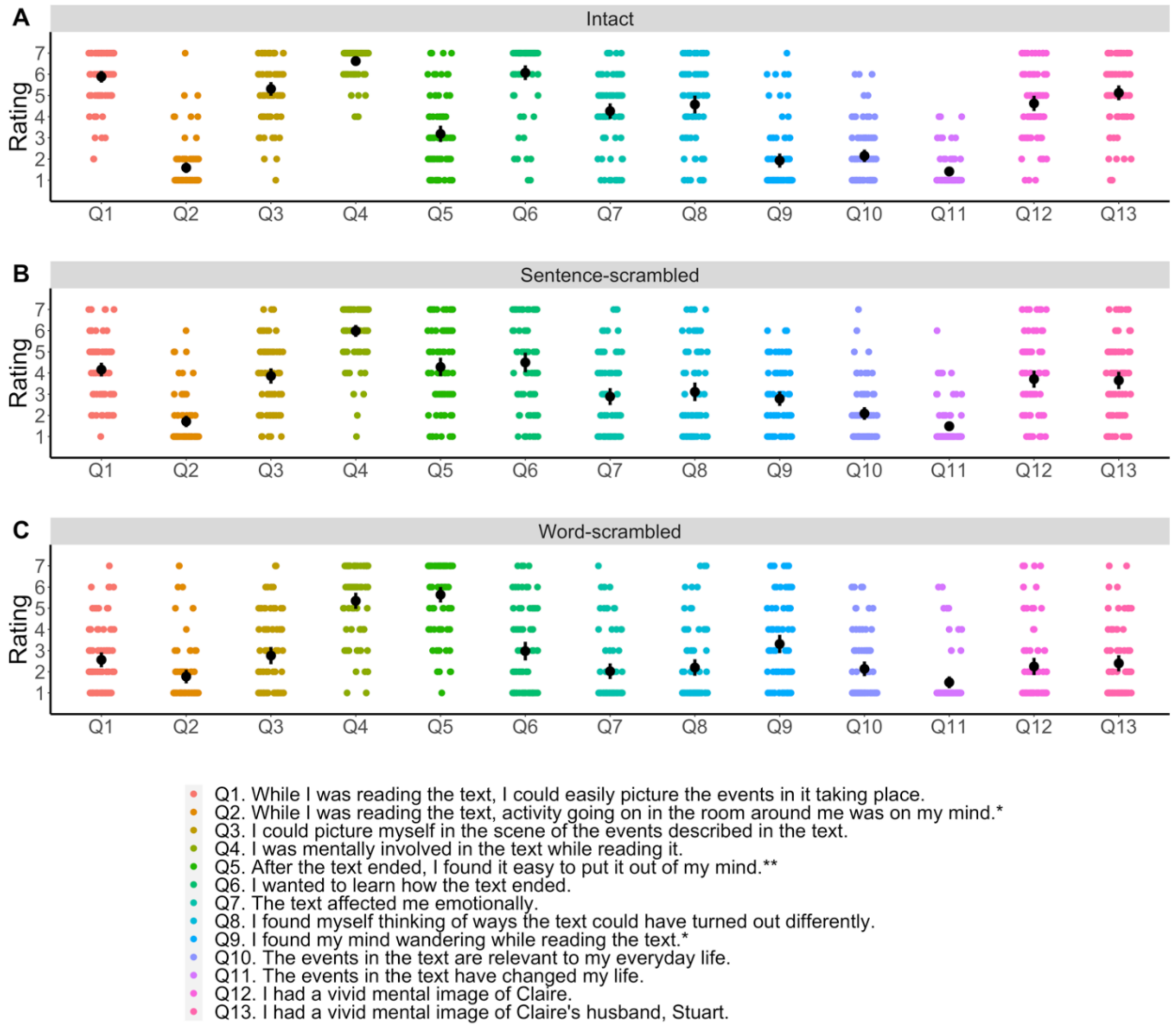

Figure S3. *Effect of scrambling on narrative transportation by item.* Distribution of transportation scores for all participants from the Carver (Original) dataset in Experiment 1. Visual inspection reveals differing profiles of transportation across conditions. Items reported here were modified from the Narrative Transportation Scale (Green & Brock, 2000). Participants provided their ratings using a 7-pint Likert scale (1 = “Not at all”, 7 = “Very much”). Each point represents a participant. Black points represent condition means. Error bars reflect 95% confidence intervals. * Represents items that were reverse scored. ** This item was reverse scored and excluded from our transportation measure due to its conceptual similarity to lingering. Data from n = 80 participants per condition. Data from each condition is plotted in a separate panel. Source data are provided as a Source Data file.

(IV) Follow-up analyses on lingering themes in post-story thoughts [Exp 1].

In the main manuscript, we directly tested the persistence of story themes on post-story spontaneous thought using a semantic analysis based on word embeddings (Global Vector embeddings; GloVe). Immediately following post-story free association, participants generated 10 words that they believed related to the central themes and ideas of the text they had read. For each story, we selected the 10 words that were mentioned most frequently across participants (for details, see Methods). We then converted each free associate and each story theme to a 300-dimensional vector using the GloVe embeddings, allowing us to estimate the semantic similarity between words. Specifically, we quantified the semantic similarity between a

participant's free association chains and the story's core themes using a metric we defined as "theme similarity": the maximum cosine similarity between a given free associate and all 10 of the story's consensus theme words (Figure 5A).

<u>*Estimating within-condition themes*</u>: One potential limitation of the original approach is that themes were estimated across conditions. However, participants who read different versions of the stories (e.g., Intact, Sentence-scrambled, Word-scrambled) may experience different themes altogether. Therefore, there is reason to consider defining the 'core themes' of a story within a given condition, rather than across. To this end, we recalculated theme similarity using themes estimated within a condition. Like the across-condition version, the 10 theme words used for a given participant's theme similarity calculation were selected by taking the most frequent theme words produced across participants, after excluding that participant's own theme words. This leave-one-participant out procedure was included to remove circularity from the analysis, however, for the majority of participants, the theme words calculated this way were identical if not highly comparable to those calculated from the full sample.

Again, using within-condition themes replicated the pattern from the main manuscript: coherence at the level of narratives, and not sentences or words, was most likely to elicit a lingering effect in which story themes shaped post-story thought (Figure S4). Across all four datasets, the change in theme similarity from pre-story to post-story covaried with the level of scrambling of the text (Three-way ANOVA of Phase [Pre/Post], Condition [Intact/Sentence-scrambled], and Story [Carver/Carver-Replication/Carver-Rewrite/July]; Phase * Condition: $F(1,632) = 17.21$, $p < 0.0001$, $\eta^2_G = 0.01$). Participants in both the Intact and Sentence-scrambled conditions showed more theme similarity post-story than pre-story, but the effect size was nearly twice as large for the Intact condition [Intact: *Pre* = 0.256, *Post* = 0.286, $t(319) = 8.98$, $p < 0.0001$, $d = 0.57$; Sentence-scrambled: *Pre* = 0.274, *Post* = 0.286, $t(319) = 4.29$, $p < 0.0001$, $d = 0.29$]. A separate paired t-test again confirmed no difference in theme similarity between pre- to post-story when the narrative was scrambled at the word-level [Word-scrambled: *Pre* = 0.272, *Post* = 0.271, $t(79) = 0.07$, $p = 0.95$, $d = 0.01$]. Therefore, our pattern of results is replicated whether themes are estimated within or across-conditions.

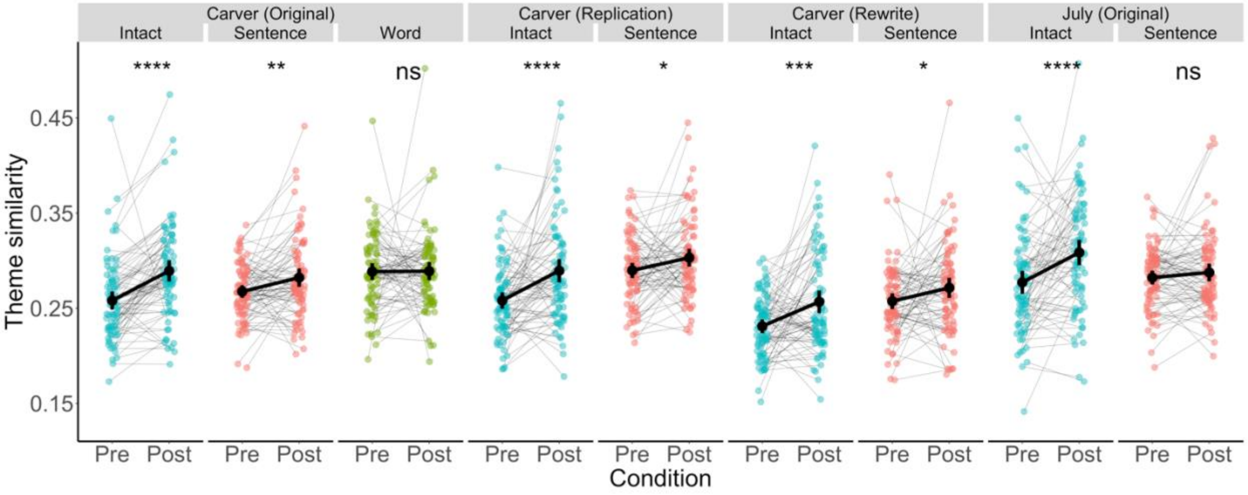

Figure S4. *Themes linger in post-story thought, irrespective of whether they are estimated within or across-conditions*. In this version of the theme similarity analysis (see Figure 5 in Manuscript), core themes are estimated for each condition separately. Theme similarity was then averaged, per participant, across all associates produced pre- and post-story. Again, story themes linger in post-story thoughts, particularly for participants in the Intact conditions. Grey lines show the change in theme similarity within-participant. Group

means are displayed using black circles. Error bars represent 95% confidence intervals. For display purposes, significance was estimated with paired-sample t-tests comparing pre- vs. post-story theme similarity [*ns* p > .05; * p =< .05; ** p =<.01, *** p =< .001, **** p =< .0001]. n = 80 participants per condition, per experiment. Source data are provided as a Source Data file.

Theme similarity is story-specific: In order to further validate our measure of theme similarity, we conducted an additional control analysis. If theme similarity is truly capturing the lingering themes from the story, we should not see a different between pre- and post-story if the core themes input into the analysis are unrelated to the story's actual themes.

To this end, repeated our theme similarity analysis using the participant-generated core themes from the July story to measure theme similarity in the Carver datasets [Carver (Original), Carver (Replication), and Carver (Rewrite)] and using the core themes from Carver (Original) to estimate theme similarity in the July dataset. Given that the Carver and July stories are about very different topics (a murder and a chance romantic encounter, respectively), we have no reason to expect the July themes to linger in mind after reading the Carver story (and vice versa).

As expected, across all four datasets, we observed no change in theme similarity from pre-story to post-story after swapping themes across stories (Figure S5; Three-way ANOVA of Phase [Pre/Post], Condition [Intact/Sentence-scrambled], and Story [Carver/Carver-Replication/Carver-Rewrite/July]; Phase * Condition: $F(1,632) = 0.26$, $p = 0.61$, $\eta^2_G = 0.0001$). A separate paired t-test further confirmed no difference in theme similarity between pre- to post-story when the narrative was scrambled at the word-level [Word-scrambled: *Pre* = 0.269, *Post* = 0.270, $t(79) = -0.28$, $p = 0.78$, $d = -0.03$]. Therefore, what lingers in post-story thought is indeed specific to the story one has just finished reading.

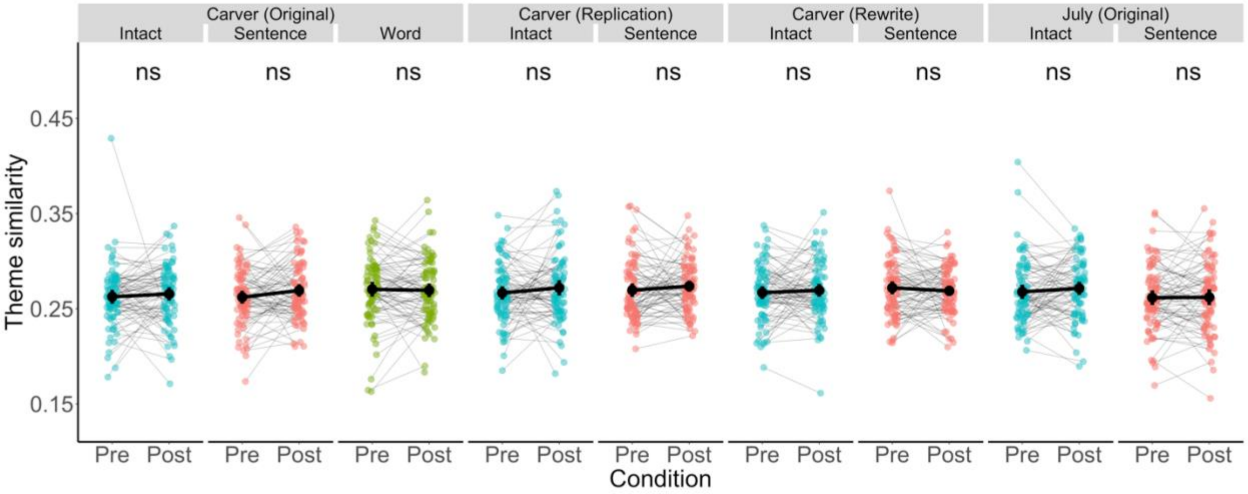

Figure S5. *Lingering themes in post-story thought are story-specific*. In this control analysis, we used the participant-generated core themes from the July story to measure theme similarity in the Carver datasets [Carver (Original), Carver (Replication), and Carver (Rewrite)]. To measure theme similarity in the July dataset, we used the core themes from Carver (Original). Theme similarity using across-story themes revealed no difference between pre- and post-story free association. Therefore, when themes linger in post-story thought, they are specific to the preceding story. Grey lines show the change in theme similarity within-participant. Group means are displayed using black circles. Error bars represent 95% confidence intervals. For display purposes, significance was estimated with paired-sample t-tests comparing pre- vs. post-story

theme similarity [*ns* p > .05; * p =< .05; ** p =<.01, *** p =< .001, **** p =< .0001]. n = 80 participants per condition, per experiment. Source data are provided as a Source Data file.

(V) Cover task performance [Exp 2].

In Experiment 2, participants encoded a list of words while performing one of four different cover tasks. Cover tasks ranged from orienting participants towards the surface-level features of the word-list (i.e., whether or not a word was italicized), to the word-level meaning (i.e., whether or not a word represented something tangible) or the list-level meaning (i.e., whether or not a word belonged to a latent theme or story). To quantify performance on the cover task, we calculated accuracy using d' per participant, or the scaled hit rate minus the scaled false alarm rate for target trials, followed by a one-sample t-test against 0 for each condition (Figure S6).

Note that, for each condition, 25% of words in the word list were targets. In the Italic and Tangible conditions, target words were italicized or tangible, respectively. In the Theme and Story conditions, target words were the unrelated decoys, as the majority of the word list (75%) was related to the Carver story.

Participants in all condition showed above chance accuracy: Italic [$M = 4.15$, $t(79) = 38.83$, $p < 0.0001$, $d = 4.34$], Tangible [$M = 2.62$, $t(79) = 22.60$, $p < 0.0001$, $d = 2.53$], Theme [$M = 1.47$, $t(79) = 9.89$, $p < 0.0001$, $d = 1.11$], and Story [$M = 1.60$, $t(79) = 10.88$, $p < 0.0001$, $d = 1.22$].

Performance in the Theme and Story conditions was considerably worse than Tangible and Italic. This is unsurprising given the difficulty of detecting a latent theme across a list of words as compared to deciding whether a word represents something tangible or if it is presented in italic type. It is important to note that some participants in the Theme and Story condition received negative d' values, suggesting they were more likely incorrectly endorse a target as related to the story/theme than correctly endorse it as an unrelated decoy. These participants were kept in the final sample as we only sought to manipulate the depth with which participants processed the words. Participants with below chance performance may have still tried to engage with across-element meaning in the word list, and thus processing the words more deeply, despite failing to learn the true story/theme.

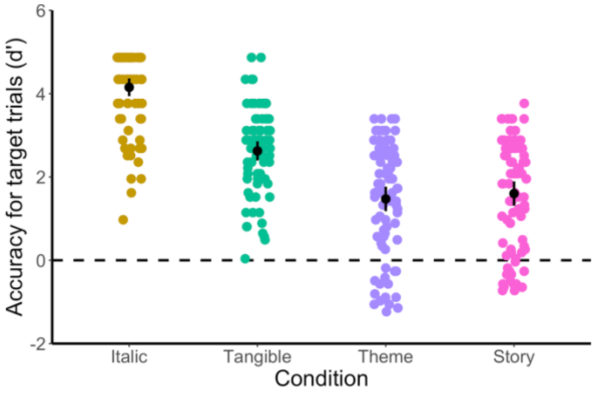

Figure S6. *Cover task performance in Experiment 2.* Likelihood of accurately detecting target trials was calculated using d' [z(hit rate) – z(false alarm rate)]. Performance was significantly above chance for each condition, though poorer for the more difficult Theme and Story tasks. Each point represents target accuracy for a participant. Black points represent condition means. Error bars reflect 95% confidence intervals. n = 80 participants per condition. Source data are provided as a Source Data file.

(VI) Validating the levels of processing manipulation with free recall [Exp 2].

To determine whether our experimental manipulation resulted in deeper processing of the incidentally studied words, we examined free recall performance. If deeper processing leads to formation of stronger memory traces, we expect better recall in deeper (e.g., Theme and Story) as compared to shallow encoding conditions (e.g., Italic and Tangible). Recall performance was quantified as the number of accurately recalled words from the 268-word study list. Free recall performance was indeed influenced by the decision participants were instructed to perform (Figure S7; One-way ANOVA of Condition [Italic/Tangible/Theme/Story], Condition: $F(3,316) = 45.26$, $p < 0.0001$, $\eta^2_G = 0.30$). Participants in the Italic condition recalled significantly fewer words (M = 5.01) than participants in the Tangible (M = 9.89, $t(158) = -5.77$, $p < 0.0001$, d = -0.91), Theme (M = 16.08, $t(158) = -10.87$, $p < 0.0001$, d = -1.72), and Story conditions (M = 18.16, $t(158) = -9.65$, $p < 0.0001$, d = -1.53). Participants in the Tangible condition also recalled significantly fewer words than participants in the Theme($t(158) = -5.40$, $p < 0.0001$, d = -0.85) and Story conditions ($t(158) = -5.67$ $p < 0.0001$, d = -0.90). Recall performance was comparable across the Theme and Story conditions ($t(158) = -1.33$, $p = 0.59$, d = -0.21).

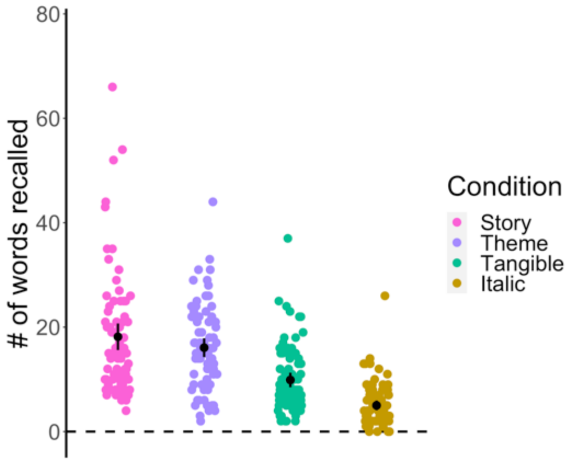

Figure S7. *Validation of depth of processing manipulation on free recall.* Distribution of words recalled from the 268-word list for all participants across conditions in Experiment 2. Conditions purported to require deeper processing (i.e., Story and Theme) were indeed associated with better free recall performance as compared to conditions requiring shallower processing (i.e., Italic and Tangible). Note that encoding was incidental as participants were not instructed that their memory for the word list would be tested. Each point represents a participant. Black points represent condition means. Error bars reflect 95% confidence intervals. n = 80 participants per condition. Source data are provided as a Source Data file.

(VII) Effects of deep processing on comprehension and transportation [Exp 2].

Comprehension of verifiable story details was operationalized as the proportion of correct responses on a 24-item multiple choice test based on the original Carver story (Figure S8). Note that participants in Experiment 2 did not read the story, but instead were presented with a list of 268 words, of which 201 of which (~75%) were related to the story and manually arranged to reflect the plot. Therefore, we expected comprehension test performance to be low overall, but relatively higher for the participants who were instructed to attend to the relationships between words (Story and Theme conditions) as compared to those who made judgments about the

words individually (i.e., Italic and Tangible conditions). A one-way (Condition: Italic, Tangible, Theme, Story) between-subjects ANOVA on comprehension produced a significant main effect of Condition [$F(3,316) = 55.01$, $p < 0.0001$, $\eta^2_G = 0.34$]. Results from paired t-tests were consistent with our predictions. Participants in the Italic condition showed less story comprehension ($M = 0.35$) than participants in the Tangible ($M = 0.52$, $t(158) = -8.72$, $p < 0.0001$, $d = -1.38$), Theme ($M = 0.57$, $t(158) = -11.03$, $p < 0.0001$, $d = -1.74$), and Story conditions ($M = 0.58$, $t(158) = -10.81$, $p < 0.0001$, $d = -1.71$). Participants in the Tangible condition also showed poorer comprehension test performance than participants in the Story condition ($t(158) = -2.84$ $p = 0.025$, $d = -0.45$) and marginally less than participants in the Theme condition ($t(158) = -2.57$, $p = 0.089$, $d = -0.41$). Recall performance was comparable across the Theme and Story conditions ($t(158) = -0.43$, $p = 1.0$, $d = -0.07$).

Narrative transportation was operationalized using a modified version of the Narrative Transportation Questionnaire (Green & Brock, 2000), including 12 items, each receiving a score of 1-7. Transportation was summarized as a proportion of the maximum attainable score (i.e., 7*12 = 84). Considering that participants did not read a story in this experiment, transportation was measured after informing all participants that the word list was not random but instead ordered to convey a hidden story. The questions in the transportation questionnaire assessed the extent to which participants were transported by this hidden story.

A one-way (Italic, Tangible, Theme, Story) between-subjects ANOVA on transportation produced a significant main effect of Condition [$F(3,316) = 83.99$, $p < 0.0001$, $\eta^2_G = 0.44$] (Figure S9). Participants in the Italic condition reported similar extents of transportation ($M = 0.39$) as participants in the Tangible ($M = 0.43$, $t(158) = -1.73$, $p = 0.29$, $d = -0.27$), but less transportation than the Theme ($M = 0.65$, $t(158) = -11.01$, $p < 0.0001$, $d = -1.74$) and Story conditions ($M = 0.58$, $t(158) = -13.31$, $p < 0.0001$, $d = -2.10$). Participants in the Tangible condition also reported less transportation than participants in the Theme ($t(158) = -9.11$ $p < 0.0001$, $d = -1.44$) and Story conditions ($t(158) = -11.23$, $p < 0.0001$, $d = -1.78$). Again, no difference was observed between Theme and Story conditions ($t(158) = -1.46$, $p = 0.146$, $d = -0.23$).

While transportation in the Theme condition was numerically lower than that of Story, this difference was not statistically significant. This point, in addition to the comparable performance in free recall (Figure S7) suggests that depth of processing may have been similar across these two conditions. In line with this account, participants in these conditions reported comparable levels of self-reported lingering and theme similarity in free association (Manuscript, Figure 6).

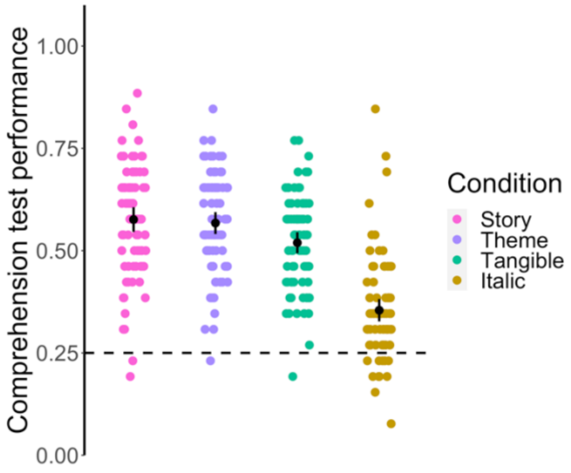

Figure S8. *Deeper processing leads to better performance on a comprehension test.* Distribution of comprehension test scores for all participants across conditions in Experiment 2. Conditions where participants encoded the list of the words more deeply (i.e., Story and Theme) was associated with better story comprehension as compared to conditions requiring shallower processing (i.e., Italic and Tangible). Comprehension of verifiable story details was operationalized as the proportion of correct responses on a 24-item multiple choice test based on the original Carver story. Note that participants in this experiment were never presented the story. Participants were presented with a list of 268 words, where 75% of the words were related to the Carver story and were manually sequenced to reflect its plot. Therefore, higher comprehension scores provide evidence that participants were able to get a sense of the underlying story, particularly in conditions where they were instructed to attend to the latent story/themes shared across words in the list. Each point represents a participant. Black points represent condition means. Error bars reflect 95% confidence intervals. Dashed horizontal line represents chance performance (each question had four response options, making chance = 25%). n = 80 participants per condition. Source data are provided as a Source Data file.

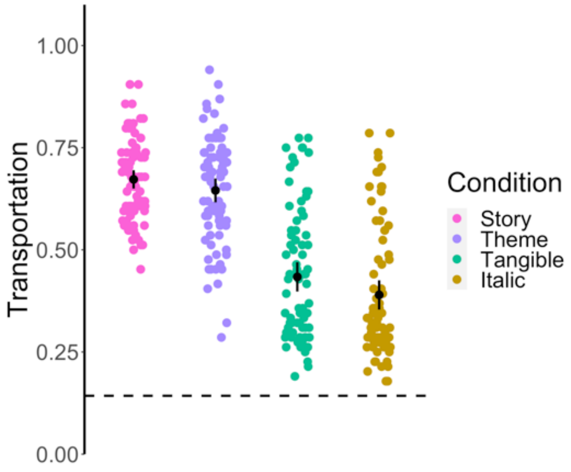

Figure S9. *Deeper processing leads to more transportation*. Distribution of transportation scores for all participants across conditions in Experiment 2. Conditions where participants encoded the list of the words more deeply (i.e., Story and Theme) were associated with more transportation into the world of the story as compared to shallow processing conditions (i.e., Italic and Tangible). Transportation was assessed using a modified version of the Narrative Transportation Scale (Green & Brock, 2000) and a proportion of the maximum attainable score (max = 7*12 = 84). Given that participants did not read a story, transportation was measured after informing participants that the word list they read contained a “hidden story”, and each transportation question referenced their subjective experience of this hidden story. Each point represents a participant. Black points represent condition means. Error bars reflect 95% confidence intervals. Dashed horizontal line represents the lowest attainable score (each question received a rating of 1 to 7; lowest

attainable score = 12/84 = 0.14). n = 80 participants per condition. Source data are provided as a Source Data file.

(VIII) Lingering is not a result of using story-related cues in free association [Exp 3].

In Experiments 1 and 2, free association began with a cue word that was related to the story. For example, for participants who read any of the version of the Carver story, free association began with either "WATER" or "BODY". For those who read the July story, free association was cued with either "PLANE" or "SECRET". The rationale behind using a story-related cue word was to amplify the likelihood of observed persistence of story themes in post-story thoughts. However, it remains unclear whether the lingering we observed only occurred because we reminded our participants of the story by using a story-related cue.

To this end, we conducted an additional preregistered experiment (Experiment 3; https://aspredicted.org/qh3dv.pdf) in which we had a new set of 80 participants read the intact version of the Carver story. Experiment 3 did not include story-related cue words for pre- or post-story free association. Instead, at the beginning of free association, participants saw the following instruction: "Enter a word to begin!".

Consistent with our predictions, lingering could be observed in the absence of story-related cues (Figure S10). In terms of self-reported lingering, a one-sample version of a Mann-Whitney U test demonstrated that median lingering indeed was different from 1 ("Not at all", or a lack of self-reported lingering; $Median_{Intact}$ = 5; U = 3160, p < 0.0001, r_{rb}= 1.0; Figure S10A). Note: the median self-reported lingering from both Carver-Original and Carver-Replication (Experiment 1) was 5 as well.

We also report evidence of lingering in our objective measures: document classification (Figure S10B) and theme similarity (Figure S10C & D). In terms of document classification, the classifier was able to discriminate between pre- and post-story chains above chance despite the absence of a story-related cue (77% classification accuracy, permutation test: p < 0.002). Similarly, theme similarity was higher post-story as compared to pre-story: *Pre* = 0.266, *Post* = 0.288, $t(79) = 3.09$, p = 0.003, d = 0.42 (Figure S10C; for the change in free association content, see Figure S12). Interestingly, theme similarity was still strongest immediately after the story ended (Figure S10D), suggesting that the timecourses of theme similarity reported in Experiment 1 (Manuscript, Figure 4B) were not an artefact of the story-related cue in free association.

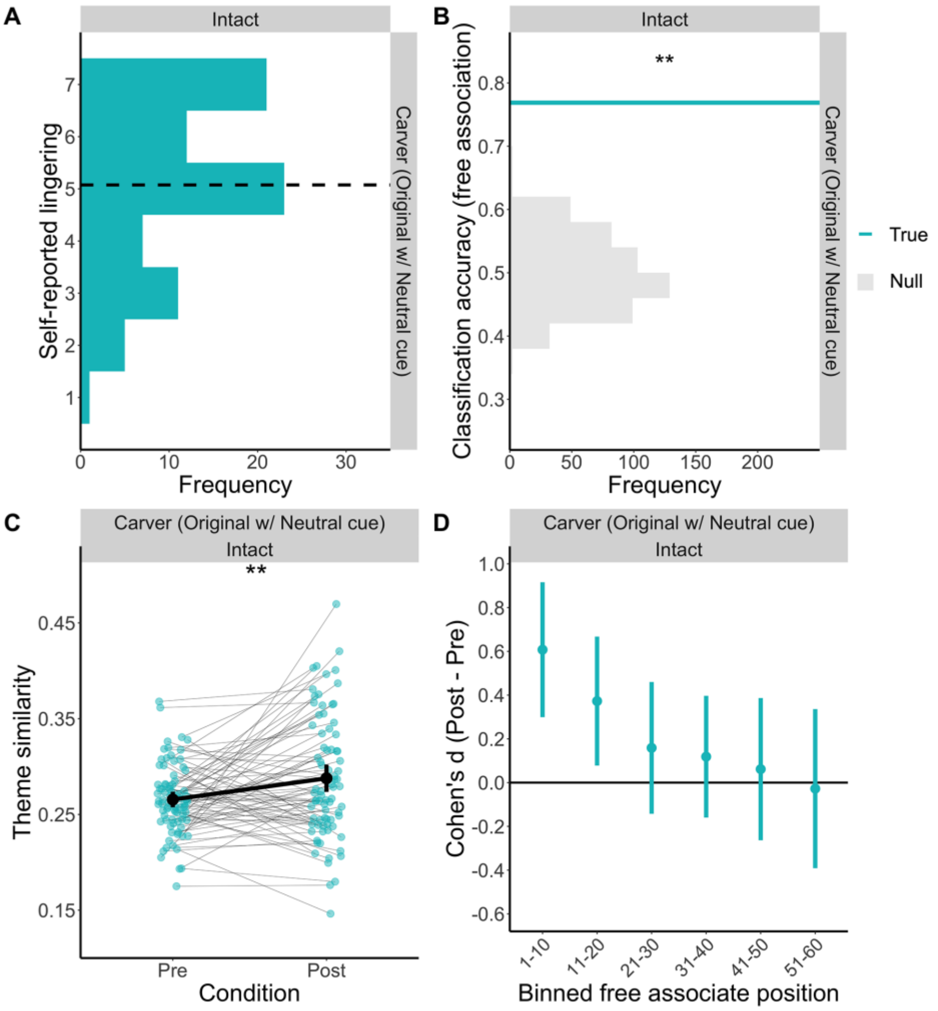

Figure S10. *Lingering is not a result of using story-related cues in free association (Experiment 3).* **(A)** Histograms revealed that participants continued to self-report the experience of lingering despite being presented with a neutral ("Enter a word to begin!") rather than a story-related cue during free association. Participants provided their rating on a 7-pt scale: 7 = very much, 1 = not at all. Black dashed line represents the mean rating per condition. n = 80 participants. **(B)** Results of document classification again indicate that pre- and post-story free association chains were discriminable. Classifiers were trained on all participants from this experiment (n = 80 participants), using a leave-one-participant-out cross-validation procedure with 500 bootstraps. Solid line represents the mean classification accuracy. Null distributions are plotted in gray. [*ns* p > .05; * p =< .05; ** p =<.01; Note 1: all *p*s are uncorrected with respect to multiple comparisons; Note 2: minimum p-value estimate for this analysis is *p* < 0.002] **C)** Theme similarity pre- and post-task highlights evidence for an increase in similarity to story themes after reading. Grey lines show the change in theme similarity within-participant. Group means are displayed using black circles. Error bars represent 95% confidence intervals. For display purposes, significance was estimated with paired-sample t-tests comparing pre- vs. post-task theme similarity [*ns* p > .05; * p =< .05; ** p =<.01, *** p =< .001, **** p =< .0001]. n = 80 participants. **(D)** Timecourse of post-story theme similarity displayed using 10-associate windows. Despite using a cue that was not directly related to the story, theme similarity was strongest immediately after the story ended. Effect size was calculated using Cohen's *d*, comparing theme similarity post-story minus pre-story within each window, and represented with a solid circle. Error bars represent 95% confidence intervals. n = 80 participants. Source data for all panels are provided as a Source Data file.

(IX) Shallow processing of a coherent story reduces lingering [Exp 4].

Experiment 1 and 2 provide evidence that a deeply processed input will linger in mind. In Experiment 1, we limited the depth with which participants could process a narrative by

scrambling the order of its constituent sentences and words. In Experiment 2, we demonstrated that deeply processing a non-narrative stimulus (i.e., a list of words) can also result in the content of that list persisting in mind. However, can we limit how deeply we process a coherent narrative? And if so, will that narrative be less likely to linger in mind?

To answer this question, we conducted an additional preregistered experiment (Experiment 4; https://aspredicted.org/xd38t.pdf) in which participants read an identical, coherent version of the Carver story, while performing different cover tasks that manipulated depth of processing.

All participants were presented with a version of the original Carver story, without scrambling. However, the text was edited such that 50% of the sentences contained one or both of the following error types: a font error or a spelling error. Participants were randomly assigned to one of two conditions, one that encouraged shallow processing of the story (i.e., Proofread) and another that encouraged deep processing (i.e., Emotion). In the Proofread condition, participants were instructed to count the number of errors in each sentence in the story. In the Emotion condition, participants were instructed to rate the emotionality of each sentence instead, while ignoring the errors.

In line with our hypothesis that deep processing enhances lingering, participants in the Emotion condition reported more lingering than participants in the Proofread condition (Figure S11A; $Median_{Emotion} = 4.5$, $Median_{Proofread} = 4$; $U = 3882$, $p = 0.018$, $r_{rb} = 0.21$). Self-report converged with our objective measures in that our document classifier was able to accurately classify free association chains as pre- or post-story for participants in the Emotion condition (64% classification accuracy, permutation test: $p = 0.008$) but not the Proofread condition (43% classification accuracy, permutation test: $p = 0.88$; S10B). Interestingly, theme similarity did not reveal a difference across conditions when averaging over the entire 5-minutes of free association (Figure S11C; Two-way ANOVA of Phase [Pre/Post] and Condition [Emotion/Proofread]; Phase * Condition: $F(1,158) = 0.46$, $p = 0.49$, $\eta^2_G = 0.002$). However, when plotting the effect size timecourse we can see a statistically significant boost in post-story theme similarity in the first bin immediately after the story ends for participants in the Emotion but not Proofread condition (Figure S11D). Also, some evidence of persistent story themes, particular to the Emotion condition, can be seen when plotting the free association bias scores (Figure S12).

One possible explanation for the relatively weak effects of theme similarity in the Emotion condition may be that the task of judging the valence of each sentence in a story actually distracts readers from engaging with its overall meaning. In other words, maybe the Emotion judgement task limits the depth with which we process the text relative to when we read a coherent story in the absence of a cover task. To this end, we directly examined whether participants who read an intact version of the Carver story [i.e., Experiment 1: Carver (Original), Carver (Replication), Carver (Rewrite); Experiment 3: Carver (Original w/ Neutral cue)] reported (i) more lingering and (ii) more transportation while reading than participants in the Emotion condition (Experiment 4).

Indeed, participants who were permitted to freely read the intact version of the Carver story reported more lingering than participants who had to judge the valence of each sentence in the story ($Median_{Intact} = 5$, $Median_{Emotion} = 4.5$; Mann-Whitney U test: $U = 10208$, $p = 0.004$, $r_{rb} = 0.20$). Similarly, participants in the Intact condition reported feeling more transported into the story while reading as compared to those in the Emotion condition ($M_{Intact} = 0.63$, $M_{Emotion} = 0.60$; Welch two-sample t-test: $t(124.33) = 2.43$, $p = 0.003$, $d = 0.30$). Given transportation can serve as a proxy for how deeply participants process the text, the Emotion condition appears to reduce depth of processing as compared to unconstrained reading of a coherent story.

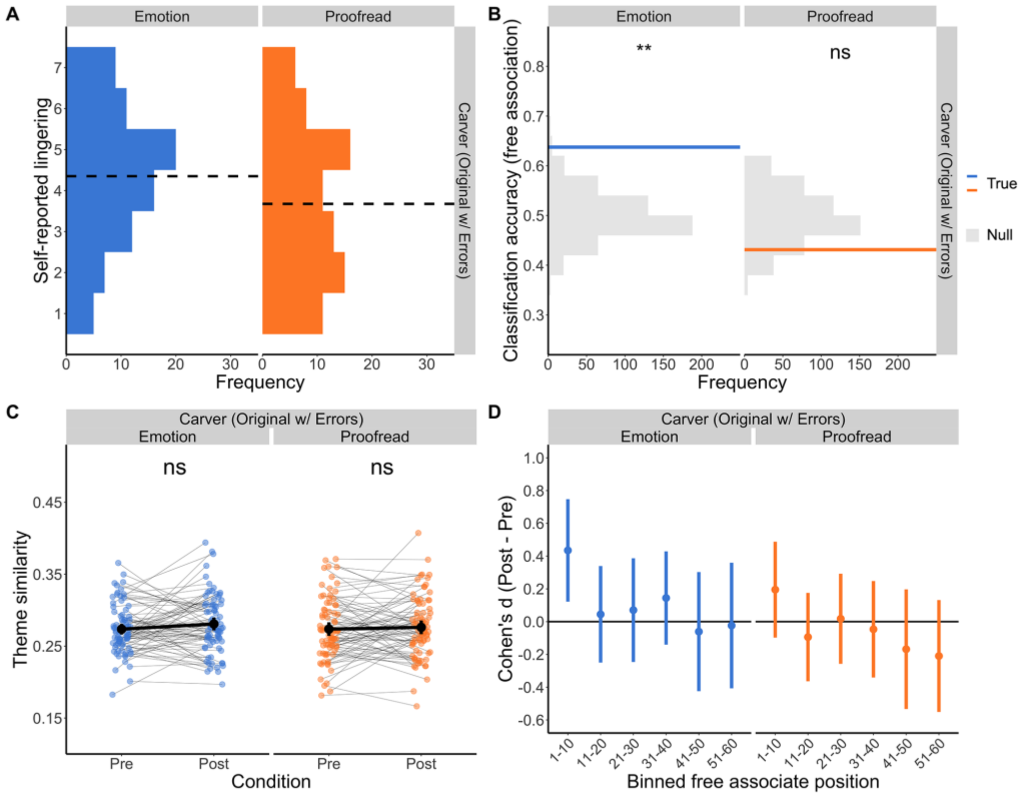

Figure S11*. Shallow processing of a coherent story reduces lingering (Experiment 4).* **(A)** Histograms revealed that participants assigned to a cover task emphasizing deep processing of the story (Emotion) reported more lingering than those assigned to a shallow reading condition (Proofread). Participants provided their rating on a 7-pt scale: 7 = very much, 1 = not at all. Black dashed line represents the mean rating per condition. n = 80 participants per condition. **(B)** Results of document classification again indicate that pre- and post-story free association chains were discriminable for participants assigned to the Emotion but not Proofread condition. Classifiers were trained on all participants from a given condition (n = 80 participants), using a leave-one-participant-out cross-validation procedure with 500 bootstraps. Solid line represents the mean classification accuracy. Null distributions are plotted in gray. [*ns* p > .05; * p =< .05; ** p =<.01; Note 1: all *p*s are uncorrected with respect to multiple comparisons; Note 2: minimum p-value estimate for this analysis is *p* < 0.002] **(C)** Theme similarity pre- and post-story fails to show any increase in the semantic closeness to the story themes after reading, for either condition. Grey lines show the change in theme similarity within-participant. Group means are displayed using black circles. Error bars represent 95% confidence intervals. For display purposes, significance was estimated with paired-sample t-tests comparing pre- vs. post-task theme similarity [*ns* p > .05; * p =< .05; ** p =<.01, *** p =< .001, **** p =< .0001]. n = 80 participants per condition. **(D)** While theme similarity averaged over all 5-minutes of free association did not show evidence of lingering, examining the timecourses of post-story theme similarity using 10-associate windows revealed evidence of increased theme similarity immediately after reading, particularly with participants in the Emotion condition. Effect size was calculated using Cohen's *d*, comparing theme similarity post-story minus pre-story within each window, and represented with a solid circle. Error bars represent 95% confidence intervals. n = 80 participants per condition. Source data for all panels are provided as a Source Data file.

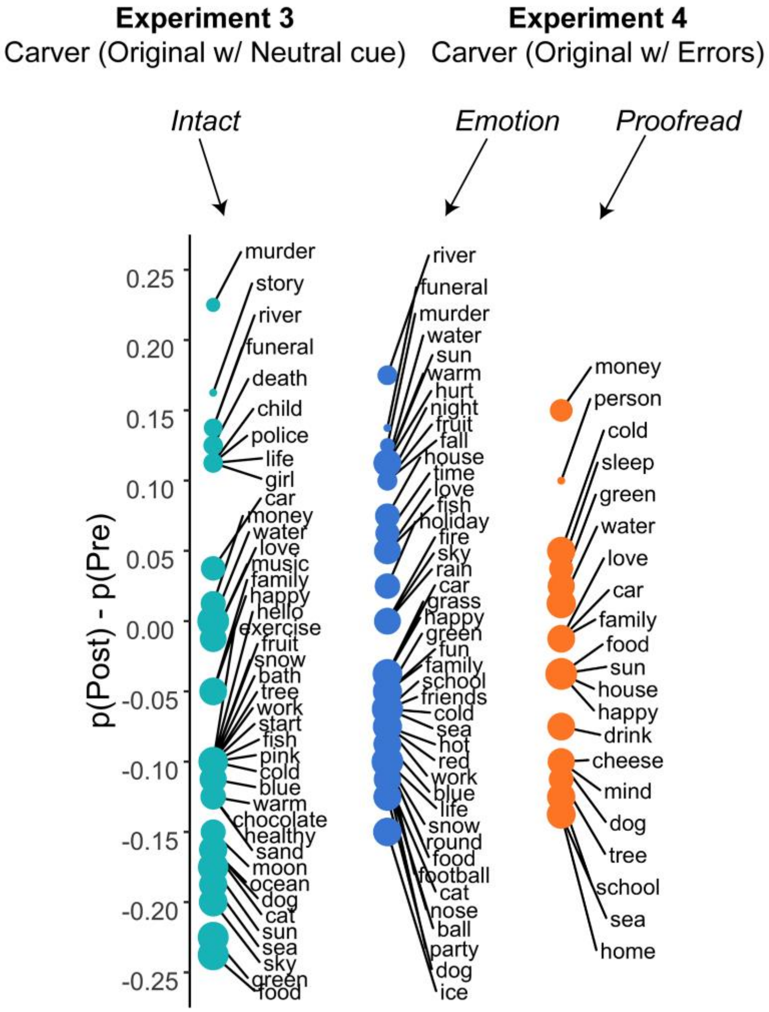

Figure S12. *Biases in free association content for Experiments 3 and 4*. Bias was defined as the proportion of post-story free association chains that contained a given word [p(Post)] minus the proportion of pre-story free association chains containing the same word [p(Pre)]. p(Post) and p(Pre) were both calculated based on the total of 80 free association chains. Therefore, positive values reflect words that are more likely to occur in post-story free association as compared to pre-story. Negative values reflect words that are more likely to occur in pre-story free association as compared to post-story. For legibility, only free associates that occurred in at least 16% of free association chains or showed a 10% bias for pre- or post-story are displayed. Size of points represents a given word's p(Pre). Source data are provided as a Source Data file.

(X) Performance on comprehension and transportation [Exp 3 & 4].

Participants in Experiment 3 obtained a mean comprehension score of 0.864 (Figure S13) and a mean transportation score of 0.627 (Figure S14).

For Experiment 4, we conducted an independent samples t-test to compare comprehension of verifiable story details and transportation across participants in the Proofread and Emotion conditions. Participants in the Proofread condition ($M = 0.70$) showed poorer performance on the comprehension test than participants in the Emotion condition ($M = 0.84$) ($t(158) = -5.23$ $p <$ 0.0001, $d = -0.83$). A similar pattern of results was observed for transportation, where participants in the Proofread condition ($M = 0.56$) were less transported by the narrative than participants in the Emotion condition ($M = 0.60$) ($t(158) = -2.54$ $p = 0.012$, $d = -0.40$).

Note that the reduced transportation for participants in the Proofread condition is consistent with the idea that the Proofreading task limited depth of processing despite the objective coherence of the text.

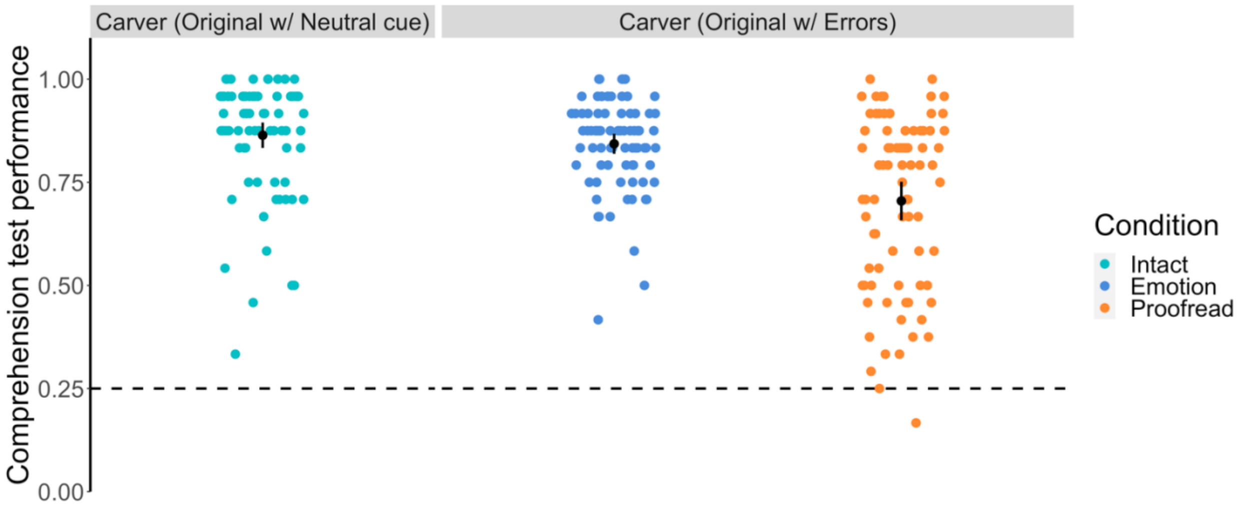

Figure S13. *Comprehension test performance for Experiments 3 and 4.* Comprehension of verifiable story details was operationalized as the proportion of correct responses on a 24-item multiple choice test based on the original Carver story. In Experiment 3 [Carver (Original w/ Neutral cue)], participants were presented with the intact version of the Carver story. In Experiment 4 [Carver (Original w/ Errors)], participants read a modified version of the Carver story where 50% of the sentence were modified to have errors in terms of inconsistent fonts or typos. Typos only occurred in 10% of sentences to maintain readability. Each point represents a participant. Black points represent condition means. Error bars reflect 95% confidence intervals. Dashed horizontal line represents chance performance (each question had four response options, making chance = 25%). n = 80 participants per condition, per experiment. Source data are provided as a Source Data file.

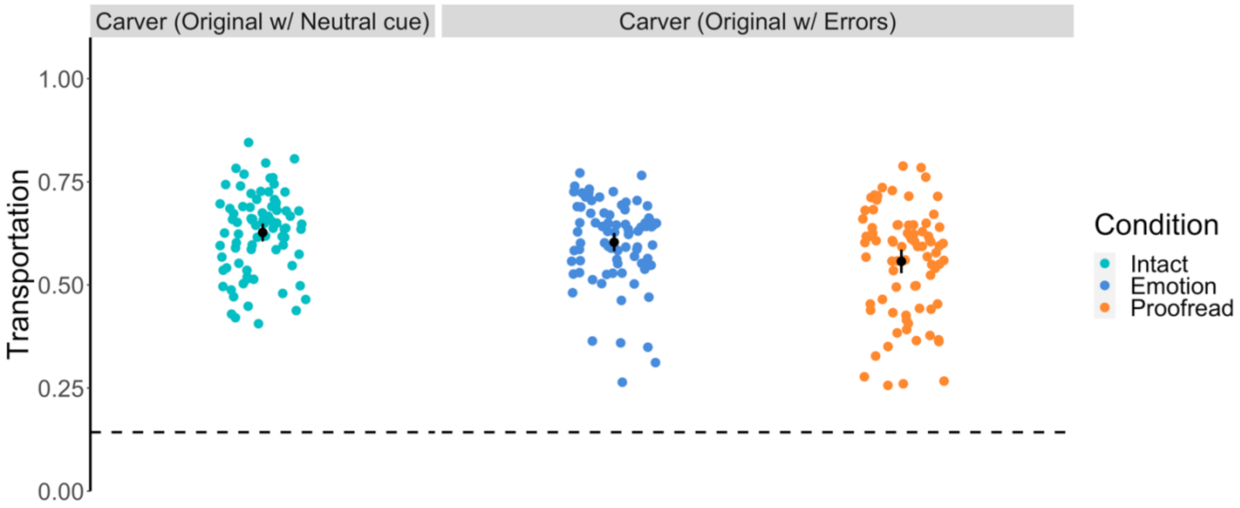

Figure S14. *Transportation for Experiments 3 and 4*. Transportation was assessed using a modified version of the Narrative Transportation Scale (Green & Brock, 2000) and a proportion of the maximum attainable

score (max = 7*12 = 84). In Experiment 3 [Carver (Original w/ Neutral cue)], participants were presented with the intact version of the Carver story. In Experiment 4 [Carver (Original w/ Errors)], participants read a modified version of the Carver story where 50% of the sentence were modified to have errors in terms of inconsistent fonts or typos. Typos only occurred in 10% of sentences to maintain readability. Each point represents a participant. Black points represent condition means. Error bars reflect 95% confidence intervals. Dashed horizontal line represents the lowest attainable score (each question received a rating of 1 to 7; lowest attainable score = 12/84 = 0.14). n = 80 participants per condition, per experiment. Source data are provided as a Source Data file.

(XI) Is lingering volitional? [Exp 3 & 4].

In Experiments 3 and 4, participants who reported experiencing lingering were additionally probed about the nature of their experience. First, participants were asked the following question: "Were you intentionally reflecting on the text while playing the word chain game? Or, did aspects of text come to mind unintentionally?" Participants had to choose one of five potential responses: (i) "I was intentionally reflecting on the text I read", (ii) "The text I read came to mind unintentionally", (iii) "Both", (iv) "Neither", (v) "Don't know". The following waffle plots display the results for Experiment 3 (Neutral cue) and Experiment 4 (Proofread vs. Emotion). Each cell represents a participant (n = 80 per condition, excluding the participants who reported a 1, i.e. "Not at all", on the self-reported lingering scale).

When combining across both experiments, we see that 51% of participants described lingering as unintentional, only 7% as intentional, 18% as both, with the remaining 24% of participants describing it as neither or unsure (see Supplemental Information). Therefore, 69% of participants reported an aspect of lingering that was outside of their volitional control.

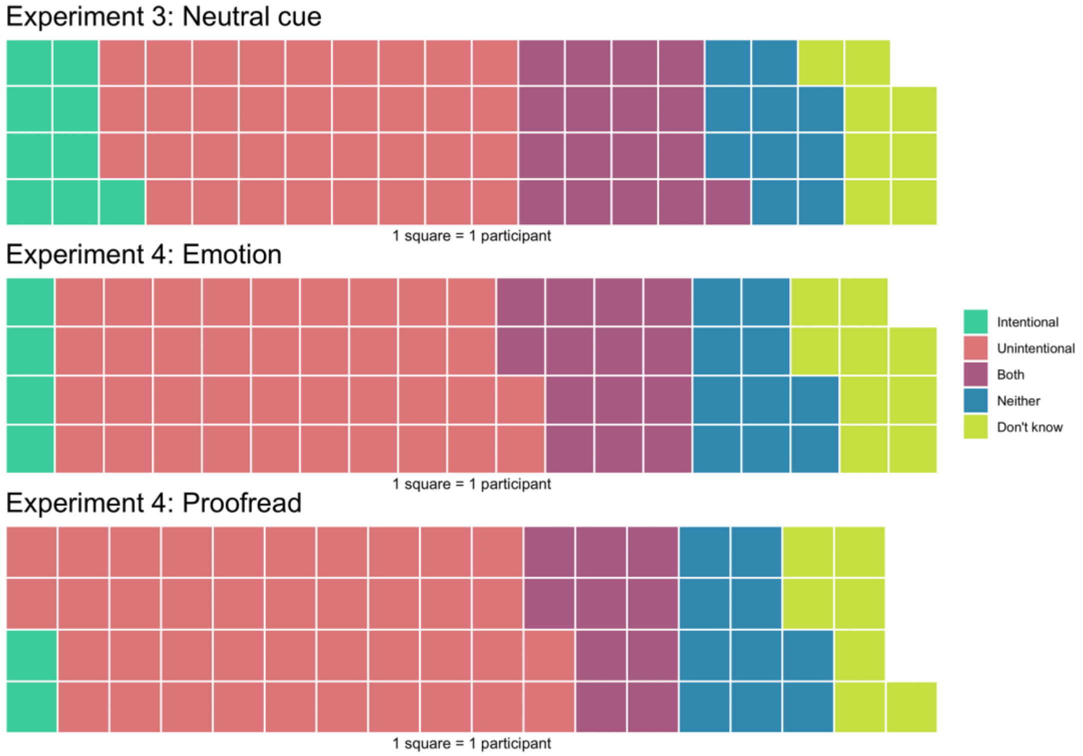

Figure S15. *Lingering may be outside of our volitional control*. Participants in Experiment 3 and 4 answered additional questions about the volitional quality of their experience of a story lingering in mind, if they experienced any lingering at all. Most participants endorsed lingering as unintentional, distinguishing it from rehearsal and the kinds of explicit memory that traditionally benefit from deep processing (Craik & Lockhart, 1972). Plots were generated in R using waffle() (Rudis, 2017). n = 80 participants per plot. Source data are provided as a Source Data file.

(XII) What does lingering feel like? [Exp 3 & 4].

In Experiments 3 and 4, participants who reported experiencing lingering were additionally probed about the nature of their experience. After the questions about the volitional quality of lingering (see above), participants were asked to describe what was lingering in their minds. Specifically, participants were asked to rate the six following statements using a 7-pt Likert scale [1 (Not At All) to 7 (Very Much)]: After reading the story, I noticed… (1) “A change in the topics that came to mind while playing the word chain game”; (2) “A change in how easy or difficult it was to come up with words while playing the word chain game”, (3) “A change in the emotions I felt while playing the word chain game”; (4) “A change in how tired I felt while playing the word chain game”; (5) “A change in how bored I felt while playing the word chain game”; and (6) There was a change in my thoughts, but it was not something that is captured by the questions above.

Overall, lingering seemed to be characterized by a change in the topics that came to mind during free association (1), the ease/difficulty they experienced when generating words (2), or a change in the emotions they felt during free association (3) (see Figure S16, S17). It was less prominently described as a change in overall boredom (4), tiredness (5) or something else altogether (6).

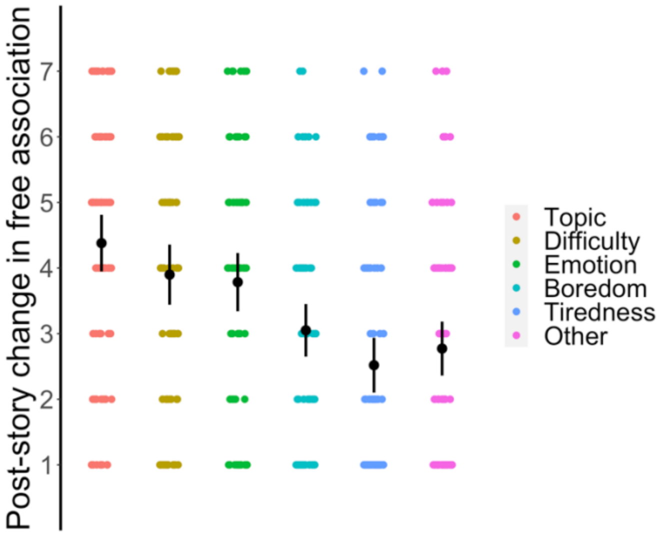

Figure S16. *Content of lingering (Experiment 3).* Participants in Experiment 3 answered additional questions about what constituted lingering in their experience. Participants tended to describe lingering as a change in the topics that came to mind during post-story free association, but also a change in the ease/difficulty of coming up with words and/or a change in the emotions they felt. Participants were less likely to describe it as boredom or tiredness. Error bars reflect 95% confidence intervals. n = 80 participants. Source data are provided as a Source Data file.

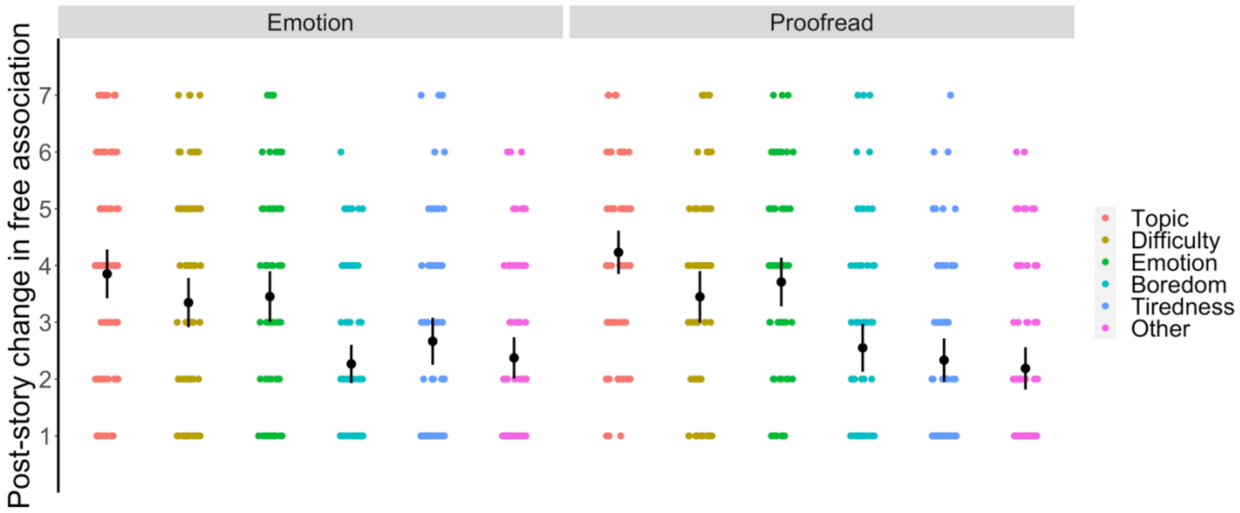

Figure S17. *Content of lingering (Experiment 4).* Participants in Experiment 4 answered additional questions about what constituted lingering in their experience. Irrespective of cover task, participants tended to describe lingering as a change in the topics that came to mind during post-story free association, but also a change in the ease/difficulty of coming up with words and/or a change in the emotions they felt. Participants were less likely to describe it as boredom or tiredness. Error bars reflect 95% confidence intervals. n = 80 participants per condition. Source data are provided as a Source Data file.

(XIII) To what extent does lingering change free association? Odds ratios [Exp 1, 2, 3 & 4].

We report that deeply processing a story (or even a list of words) can results in detectable changes in the content of subsequent free association. However, despite these changes being statistically significant, how sizeable was the change in more concrete terms? To this end, we report the odds ratios for the top-20 words from pre- and post- story free association, for all conditions and all experiments.

Odds ratios for pre-story were calculated as: p(Pre)/p(Post). Similarly, odds ratios for post-story were calculated as: p(Post)/p(Pre). In other words, a post-story odds ratio of 2 would mean that a given word was twice as likely to occur in a free association chain pre-story as compared to post-story.

Overall, we can see that post-story free association was prominently shaped by the content of the text that participants read (Figure S18), particularly in conditions where the situation-level meaning was more readily available (e.g., Intact as compared to Word-scrambled). For example, words related to Raymond Carver's *So Much Water So Close to Home* such as "river", "murder", "funeral", "camping", and "dead" were more prominent post-story. While the content of post-story free association shifted to words such as "loss", "secret", "number", "spy" and "four" after participants read Miranda July's *Roy Spivey*. Therefore, content related to the story seemed to persist and exert a strong constraint over the content of post-story thoughts.

While odds are easily interpreted and can help convey the magnitude of the observed changes in free association, they can be highly sensitive to outliers, which can be an issue particularly in the context of sparse data like free association. For complementary results, see our bias scores in Figures 3 (Experiment 1), 6C (Experiment 2) and S12 (Experiments 3 and 4).

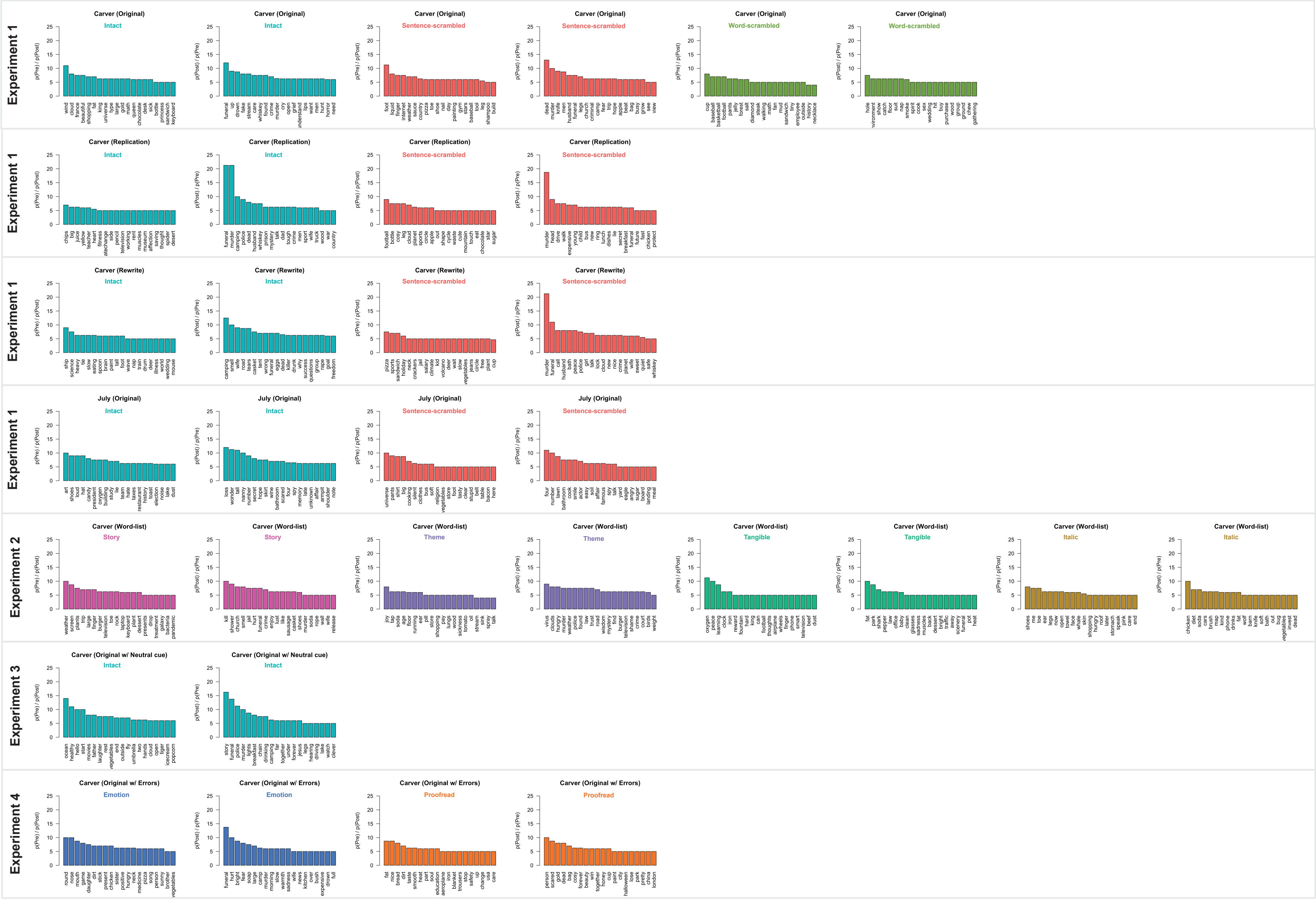

Figure S18. *Expressing changes between pre- and post-story free association in terms of odds ratios.* Odds ratios for pre-story were calculated as:

was twice as likely to occur in a free association chain post-story as compared to pre-story. n = 80 participants per condition, per experiment. Source data for all panels are provided as a Source Data file.

(XIV) Which aspects of transportation best predict lingering? [Exp 1, 3 & 4].

Transportation is a multidimensional construct, and the present operationalization (Green & Brock, 2000) consists of 13 separate items. To determine which aspects of transportation best predict the extent to which an experience lingers in mind, we input each individual item from the transportation scale as predictors in a 10-fold cross-validated regression model predicting self-reported lingering. As in the manuscript, we incorporated backwards stepwise feature selection, however, since our focus here is to determine the specific items that are most predictive of lingering, we limited the model's search space to models with a maximum of 5-predictors. We performed separate regression models for each condition in Experiments 1, 3 and 4. We did not include the data from Experiment 2, as the transportation scale was considerably modified to accommodate the word-list experiment. Specifically, after completing the task, participants were informed that the word-list contained a "hidden story" and were asked to indicate the extent to which they were immersed in it (e.g., "I could picture myself in the scene of the events described in the hidden story"). However, given the difference between these items and those in the other conditions, in addition to our uncertainty regarding how participants interpreted these questions, we chose not to include them in this item-wise analysis. All predictors and outcome variables were z-scored prior to their inclusion in the model.

The final model for each analysis is summarized below in Table S1. All models were statistically significant (ps < 0.0001) and accounted for between 17-59% of the variance in self-reported lingering across all datasets. In ten of 12 of our datasets, either Questions 6 or 7 were the best predictors of self-reported lingering. Question 6 was "I wanted to learn how the text ended", while Question 7 was "The text affected me emotionally". This highlights an interesting possibility of multiple routes to narrative transportation (and consequently, lingering): one via curiosity and the other via emotion. The full text for each question on the transportation scale can be found in Figure S3.

Note that Question 5 from the transportation scale appears to tap into the inverse of lingering (i.e., "After the text ended, I found it easy to put out of my mind"). As such, it was excluded from our calculations of transportation and from this analysis. However, the Pearson correlation between self-reported lingering and Question 5 can be found in Table S1, under the column titled "r(lingering, Q5)".

Table S1. *Summary of cross-validated backwards stepwise feature selection using individual transportation items to predict self-reported lingering*. *B* represents the standardized beta coefficients in the final model. Only regressors that were included in the final model for each dataset are displayed here. The full text for each question on the transportation scale can be found in Figure S3.

Experiment	Story	Condition	r(lingering,Q5)	Final R^2	Predictor 1	Predictor 2	Predictor 3	Predictor 4	Predictor 5
Exp 1	Carver-Original	Intact	-0.69	0.46	Q7 [*B*=0.68]	-	-	-	-
Exp 1		Sentence	-0.70	0.52	Q6 [*B*=0.53]	Q3 [*B*=0.32]	-	-	-
Exp 1		Word	-0.56	0.57	Q6 [*B*=0.32]	Q7 [*B*=0.26]	Q12 [*B*=0.17]	Q10 [*B*=0.16]	-
Exp 1	Carver-Replication	Intact	-0.71	0.44	Q7 [*B*=0.31]	Q6 [*B*=0.28]	Q10 [*B*=-0.27]	Q11 [*B*=0.23]	Q12 [*B*=0.22]
Exp 1		Sentence	-0.57	0.35	Q6 [*B*=0.60]	-	-	-	-
Exp 1	Carver-Rewrite	Intact	-0.65	0.17	Q13 [*B*=0.43]	-	-	-	-
Exp 1		Sentence	-0.68	0.38	Q6 [*B*=0.33]	Q13 [*B*=0.25]	Q7 [*B*=0.23]	-	-
Exp 1	July-Original	Intact	-0.57	0.53	Q7 [*B*=0.45]	Q1 [*B*=0.42]	-	-	-
Exp 1		Sentence	-0.56	0.46	Q8 [*B*=0.42]	Q1 [*B*=0.41]	-	-	-
Exp 3	Carver-Original (w/ Neutral cue)	Intact	-0.45	0.30	Q7 [*B*=0.56]	-	-	-	-
Exp 4	Carver-Original (w/ Edits)	Emotion	-0.63	0.25	Q7 [*B*=0.36]	Q12 [*B*=0.25]	-	-	-
Exp 4		Proofread	-0.59	0.59	Q6 [*B*=0.46]	Q1 [*B*=0.27]	Q7 [*B*=0.23]	Q10 [*B*=0.21]	-

(XV) Cross-story classification as evidence that lingering is story-specific [Exp 1 & 3].

Throughout the manuscript and supplement, we argue that lingering in free association is story specific (see Supplemental Results IV or Figure 3 in Manuscript): the content persisting in free association is yoked to content from the text that participants had just read. To further test this claim, we ran a cross-story classifier. Specifically, we hypothesized that a classifier trained on Carver-Original should be able to predict whether a free association chain was generated pre- or post-story with participants who read a different version of the same story (i.e., Carver-Rewrite). We similarly hypothesized that a classifier trained on Carver-Original should not exceed chance classification if it was tested on participants who read a different story altogether (i.e., July).

As described in the main manuscript (see Methods), we trained a linear support vector machine classifier to predict whether a given free association chain was generated before or after reading. The model was trained on data from participants in Experiment 3, who read an intact version of Carver-Original. The model was then tested on participants in from the intact conditions of Carver-Rewrite and July in Experiment 1. We decided to use the data from Experiment 3 as our training set as participants in this condition were not presented story-related cues in free association. Therefore, any above chance classification achieved in the Carver-Rewrite dataset would not be due to the shared cue words between the training and test sets.

Broadly, the input to the model was a vector of word counts from a single word-chain, indicating the number of times each unique word from all free association chains was mentioned in that chain. The output of the model was a binary prediction of whether the word-chain was "pre-story" or "post-story". Classification accuracy was the proportion of correct classifications across all free association chains (chance level = 50%; for details on null distribution see Figure S19).

Consistent with our predictions, the classifier was able to discriminate between pre- and post-story chains above chance for participants from the Carver-Rewrite dataset (58% classification accuracy, permutation test: $p = 0.002$) but not the July dataset (53% accuracy, $p = 0.17$) (Figure S19). These results are consistent with the claim that lingering is associated with the persistence of story-specific content.

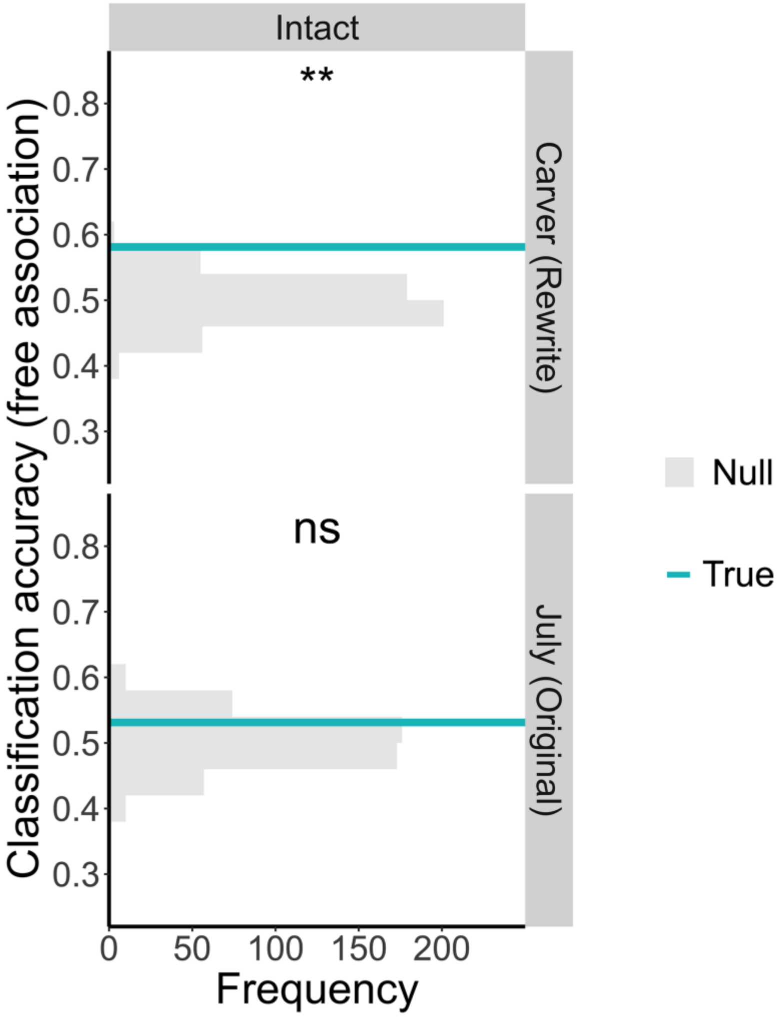

Figure S19. *Cross-story classification as evidence that lingering is story-specific.* Histograms of how accurately a document classifier could discriminate between pre- and post-story free association chains across separate datasets. Classifiers were trained on data from participants who read the Intact version of Carver-Original in Experiment 3 (Neutral cue variant; n = 80 participants). Classifiers were then tested on a separate group of participants who read a different version of the same story (i.e., Intact version of Carver-Rewrite; n = 80 participants) or a different story entirely (i.e., Intact version of July; n = 80 participants). Solid lines represent the mean classification accuracy. Null distributions were estimated by randomly shuffling the labels of word chains (pre, post) in the test set and recalculating classification accuracy over 500 permutations. Likelihood of achieving mean classification from the null distribution was calculated using a permutation test [*ns* p > .05; * p =< .05; ** p =<.01; *Note*: minimum p-value estimate for this analysis is *p* < 0.002]. Source data are provided as a Source Data file.

(XVI) Direct tests of pre- vs post-task theme similarity [Exp 1, 2, 3 & 4].

In Figure 4A, 6D, S4, S5, S10C, & S11C, asterisks were used to denote the significance of uncorrected, two-sided paired t-tests comparing theme similarity calculated pre- vs. post-story, for each condition in each dataset. These t-tests were intended only to visually complement the results of the relevant omnibus ANOVAs reported in the main manuscript and supplemental information. For the interested reader, the statistical details for each of these t-tests are reported below.

Figure 4A (Experiment 1):

Carver-Original: Paired t-tests indicate that theme similarity showed a greater post-story increase for participants in the Intact condition [$t(79) = 4.82$, $p < 0.001$, $d = 0.60$] as compared to those in the Sentence-scrambled [$t(79) = 2.77$, $p = 0.007$, $d = 0.37$] or Word-scrambled conditions [$t(79) = -0.05$, $p = 0.959$, $d = -0.01$].

Carver-Replication: Paired t-tests indicate that theme similarity showed a greater post-story increase for participants in the Intact condition [$t(79) = 4.14$, $p < 0.001$, $d = 0.59$] as compared to those in the Sentence-scrambled condition [$t(79) = 2.68$, $p = 0.009$, $d = 0.39$].

Carver-Rewrite: Paired t-tests indicate that theme similarity showed a greater post-story increase for participants in the Intact condition [$t(79) = 3.75$, $p < 0.001$, $d = 0.55$] as compared to those in the Sentence-scrambled condition [$t(79) = 1.90$, $p = 0.061$, $d = 0.29$].

July-Original: Paired t-tests indicate that theme similarity showed a greater post-story increase for participants in the Intact condition [$t(79) = 4.36$, $p < 0.001$, $d = 0.57$] as compared to those in the Sentence-scrambled condition [$t(79) = 0.85$, $p = 0.397$, $d = 0.10$].

Figure 6D (Experiment 2):

Paired t-tests indicated that participants in both the Story and Theme conditions showed more theme similarity post-task as compared to pre-, which was not true for participants in the Italic or Tangible conditions: Italic [$t(79) = -1.30$, $p = 0.200$, $d = -0.15$]; Tangible [$t(79) = 1.67$, $p = 0.100$, $d = 0.20$]; Theme [$t(79) = 4.06$, $p = 0.009$, $d = 0.32$]; Story [$t(79) = 2.51$, $p = 0.014$, $d = 0.32$].

Figure S4 (Experiment 1 – supplemental analysis: within-condition theme similarity):

Carver-Original: Paired t-tests indicate that theme similarity showed a greater post-story increase for participants in the Intact condition [$t(79) = 5.81$, $p < 0.001$, $d = 0.66$] as compared to those in the Sentence-scrambled [$t(79) = 2.99$, $p = 0.004$, $d = 0.39$] or Word-scrambled conditions [$t(79) = 0.07$, $p = 0.945$, $d = 0.01$].

Carver-Replication: Paired t-tests indicate that theme similarity showed a greater post-story increase for participants in the Intact condition [$t(79) = 4.53$, $p < 0.001$, $d = 0.65$] as compared to those in the Sentence-scrambled condition [$t(79) = 2.30$, $p = 0.024$, $d = 0.34$].

Carver-Rewrite: Paired t-tests indicate that theme similarity showed a greater post-story increase for participants in the Intact condition [$t(79) = 3.71$, $p < 0.001$, $d = 0.58$] as compared to those in the Sentence-scrambled condition [$t(79) = 2.11$, $p = 0.038$, $d = 0.33$].

July-Original: Paired t-tests indicate that theme similarity showed a greater post-story increase for participants in the Intact condition [$t(79) = 4.26$, $p < 0.001$, $d = 0.55$] as compared to those in the Sentence-scrambled condition [$t(79) = 1.18$, $p = 0.241$, $d = 0.14$].

Figure S5 (Experiment 1 – supplemental analysis: cross-story theme similarity):

Carver-Original: Paired t-tests indicate that theme similarity did not differ between pre- and post-story for participants in the Intact [$t(79) = 1.04$, $p = 0.303$, $d = 0.14$], Sentence-scrambled [$t(79) = 1.50$, $p = 0.138$, $d = 0.19$] or Word-scrambled conditions [$t(79) = -0.192$, $p = 0.848$, $d = -0.02$].

Carver-Replication: Paired t-tests indicate that theme similarity did not differ between pre- and post-story for participants in the Intact [$t(79) = 1.97$, $p = 0.056$, $d = 0.25$] or Sentence-scrambled conditions [$t(79) = 1.32$, $p = 0.195$, $d = 0.18$].

Carver-Rewrite: Paired t-tests indicate that theme similarity did not differ between pre- and post-story for participants in the Intact [$t(79) = 1.57$, $p = 0.125$, $d = 0.21$] or Sentence-scrambled conditions [$t(79) = 0.42$, $p = 0.679$, $d = 0.06$].

July-Original: Paired t-tests indicate that theme similarity did not differ between pre- and post-story for participants in the I Intact [$t(79) = 0.943$, $p = 0.348$, $d = 0.11$] or Sentence-scrambled conditions [$t(79) = 0.116$, $p = 0.908$, $d = 0.01$.

Figure S10C (Experiment 3):

Paired t-tests indicated that participants in the Intact condition showed a significant increase in theme similarity post- as compared to pre-story [$t(79) = 3.09$, $p = 0.003$, $d = 0.42$].

Figure S11C (Experiment 4):

Paired t-tests indicate that theme similarity did not differ between pre- and post-story for participants in the Emotion condition [$t(79) = 1.47$, $p = 0.146$, $d = 0.19$] or the Proofread condition [$t(79) = 0.49$, $p = 0.628$, $d = 0.06$].

Supplemental References

1. Green, M. C. & Brock, T. C. The role of transportation in the persuasiveness of public narratives. *Journal of Personality and Social Psychology* **79**, 701–721 (2000).
2. Kuijpers, M. M., Hakemulder, F., Tan, E. S. & Doicaru, M. M. Exploring absorbing reading experiences: Developing and validating a self-report scale to measure story world absorption. *Scientific Study of Literature* **4**, 89–122 (2014).
3. Carver, R. So much water so close to home. *Furious Seasons and Other Stories* 41–61 (1977).
4. July, M. Roy Spivey. *The New Yorker* (2007).
5. Chmielewski, M. & Kucker, S. C. An MTurk Crisis? Shifts in Data Quality and the Impact on Study Results. *Social Psychological and Personality Science* **11**, 464–473 (2020).
6. Nguyen, D. Q., Nguyen, D. Q., Pham, D. D. & Pham, S. B. RDRPOSTagger: A Ripple Down Rules-based Part-Of-Speech Tagger. in *Proceedings of the Demonstrations at the 14th Conference of the European Chapter of the Association for Computational Linguistics* 17–20 (Association for Computational Linguistics, 2014). doi:10.3115/v1/E14-2005.
7. Feinerer, I. & Hornik, K. tm: Text Mining Package. (2020).
8. Meyer, D., Dimitriadou, E., Hornik, K., Weingessel, A. & Leisch, F. e1071: Misc Functions of the Department of Statistics, Probability Theory Group (Formerly: E1071), TU Wien. (2019).
9. Pennington, J., Socher, R. & Manning, C. Glove: Global Vectors for Word Representation. *Proceedings of the 2014 Conference on Empirical Methods in Natural Language Processing (EMNLP)* 1532–1543 (2014) doi:10.3115/v1/D14-1162.
10. Harris, Z. S. Distributional Structure. *WORD* **10**, 146–162 (1954).
11. Torchiano, M. effsize: Efficient Effect Size Computation. (2020) doi:10.5281/zenodo.1480624.

12. Brysbaert, M., Warriner, A. B. & Kuperman, V. Concreteness ratings for 40 thousand generally known English word lemmas. *Behavior Research Methods* **46**, 904–911 (2014).
13. Warriner, A. B., Kuperman, V. & Brysbaert, M. Norms of valence, arousal, and dominance for 13,915 English lemmas. *Behavior Research Methods* **45**, 1191–1207 (2013).
14. Lovibond, P. F. & Lovibond, S. H. The structure of negative emotional states: Comparison of the Depression Anxiety Stress Scales (DASS) with the Beck Depression and Anxiety Inventories. *Behaviour Research and Therapy* **33**, 335–343 (1995).
15. Treynor, W., Gonzalez, R. & Nolen-Hoeksema, S. Rumination Reconsidered: A Psychometric Analysis. *Cognitive Therapy and Research* **27**, 247–259 (2003).
