# Supplementary material for: Narrative thinking lingers in spontaneous thought
Source: Nat Commun. 2022 Aug 6;13:4585. doi: 10.1038/s41467-022-32113-6 (PMC9357042; doi:10.1038/s41467-022-32113-6)
Supplement: Supplementary file 3 — Reporting Summary [file 41467_2022_32113_MOESM3_ESM.pdf]

nature portfolio

Corresponding author(s): Buddhika Bellana
Christopher J. Honey

Last updated by author(s): Jul 4, 2022

Reporting Summary

Nature Portfolio wishes to improve the reproducibility of the work that we publish. This form provides structure for consistency and transparency in reporting. For further information on Nature Portfolio policies, see our Editorial Policies and the Editorial Policy Checklist.

Statistics

For all statistical analyses, confirm that the following items are present in the figure legend, table legend, main text, or Methods section.

n/a	Confirmed	
☐	☒	The exact sample size (*n*) for each experimental group/condition, given as a discrete number and unit of measurement
☐	☒	A statement on whether measurements were taken from distinct samples or whether the same sample was measured repeatedly
☐	☒	The statistical test(s) used AND whether they are one- or two-sided<br>*Only common tests should be described solely by name; describe more complex techniques in the Methods section.*
☐	☒	A description of all covariates tested
☐	☒	A description of any assumptions or corrections, such as tests of normality and adjustment for multiple comparisons
☐	☒	A full description of the statistical parameters including central tendency (e.g. means) or other basic estimates (e.g. regression coefficient) AND variation (e.g. standard deviation) or associated estimates of uncertainty (e.g. confidence intervals)
☐	☒	For null hypothesis testing, the test statistic (e.g. *F*, *t*, *r*) with confidence intervals, effect sizes, degrees of freedom and *P* value noted<br>*Give P values as exact values whenever suitable.*
☒	☐	For Bayesian analysis, information on the choice of priors and Markov chain Monte Carlo settings
☒	☐	For hierarchical and complex designs, identification of the appropriate level for tests and full reporting of outcomes
☐	☒	Estimates of effect sizes (e.g. Cohen's *d*, Pearson's *r*), indicating how they were calculated

Our web collection on statistics for biologists contains articles on many of the points above.

Software and code

Policy information about availability of computer code

Data collection	Data were collected on Amazon Mechanical Turk and Prolific using the Psiturk platform (https://psiturk.org/; version: Psiturk 2), an online, open source, software for developing web experiments. Code for individual HTML components of web experiment are publicly available on Open Science Framework: https://osf.io/dmbx4/.
Data analysis	Data analysis was conducted using R Studio (https://www.rstudio.com/, version: 1.1.463), running R (https://www.r-project.org/, version: 3.6.3). R is an open source language for statistical computing. Pretrained word embeddings (GLoVe; version: Wikipedia 2014 + Gigaword 5) used in the manuscript are also publicly available via Rhe Stanford Natural Language Processing Group (https://nlp.stanford.edu/projects/glove/). RDRPOStagger (https://aclanthology.org/E14-2005/; version: 1.1) was used in R for part-of-speech tagging. tm (https://www.jstatsoft.org/article/view/v025i05, version: 0.7-7) was used in R to calculate document term matrices. e1071 (https://cran.r-project.org/web/packages/e1071/index.html; version: 1.7-3) was used in R to run the support vector machine classifiers. caret (https://cran.r-project.org/web/packages/caret/; version: 6.0-86) was used in R to run cross-validated stepwise backwards feature selection. effsize (https://cran.r-project.org/web/packages/effsize/index.html; version: 0.7.9) was used in R to calculate effect sizes.. Analysis code is available on Open Science Framework: https://osf.io/dmbx4/

For manuscripts utilizing custom algorithms or software that are central to the research but not yet described in published literature, software must be made available to editors and reviewers. We strongly encourage code deposition in a community repository (e.g. GitHub). See the Nature Portfolio guidelines for submitting code & software for further information.

Data

Policy information about availability of data

All manuscripts must include a data availability statement. This statement should provide the following information, where applicable:

- Accession codes, unique identifiers, or web links for publicly available datasets
- A description of any restrictions on data availability
- For clinical datasets or third party data, please ensure that the statement adheres to our policy

Data Availability. All data and materials from this study are available on Open Science Framework (https://osf.io/dmbx4/). Data used to generate each figure in the manuscript and supplement are provided in the Supplementary Information/Source Data file.

Code Availability. Sample analysis code is available on Open Science Framework (https://osf.io/dmbx4/).

Bellana, B., Mahabal, A. & Honey, C. J. Supplemental Information for 'Narrative thinking lingers in spontaneous thought'. (2022) doi:10.17605/OSF.IO/DMBX4.

Human research participants

Policy information about studies involving human research participants and Sex and Gender in Research.

Reporting on sex and gender

Gender self-report data was collected for each participant in all four experiments. Total counts are listed below:

Experiment 1:
After exclusions, a total of 720 participants were included in the final sample (Nmale = 360; Nfemale = 354, with 6 participants selecting "None of the above / Prefer not to identify").

Experiment 2:
After exclusions, a total of 320 participants were included in the final sample (Nmale = 201; Nfemale = 113, with 6 participants selecting "None of the above / Prefer not to identify").

Experiment 3:
After exclusions, a total of 80 participants were included in the final sample (Nmale = 17; Nfemale = 63).

Experiment 4:
After exclusions, a total of 160 participants were included in the final sample (Nmale = 76; Nfemale = 80, with 4 participants selecting "None of the above / Prefer not to identify").

We had no a priori hypotheses about gender differences in lingering and therefore did not conduct any analyses with respect to gender.

Population characteristics

See below.

Recruitment

Participants were recruited using popular online web recruitment platforms: Amazon Mechanical Turk and Prolific. Online experiments present some self-selection bias, as individuals who are interested in performing psychology experiments will be the ones most likely to participate. The present experiment was advertised using the title "Assorted Cognitive Tasks" to reduce self-selection in terms of avid readers, and therefore, we believe these results should be an acceptable sample to examine cognitive performance in adult humans.

Ethics oversight

This study protocol was approved by the Johns Hopkins University Homewood Institutional Review Board.

Note that full information on the approval of the study protocol must also be provided in the manuscript.

Field-specific reporting

Please select the one below that is the best fit for your research. If you are not sure, read the appropriate sections before making your selection.

☐ Life sciences ☒ Behavioural & social sciences ☐ Ecological, evolutionary & environmental sciences

For a reference copy of the document with all sections, see nature.com/documents/nr-reporting-summary-flat.pdf

Behavioural & social sciences study design

All studies must disclose on these points even when the disclosure is negative.

Study description	Mixed-methods experimental study on why certain kinds of experiences linger in our spontaneous thoughts.
Research sample	Participants age 18 and older living in the USA/UK were recruited via Amazon Mechanical Turk (AMT) and Prolific. Participants on AMT/Prolific have been found to be reasonably representative of the general population along psychological dimensions, but tend to have somewhat higher negative affect and lower social engagement (McCredie & Morey, 2018).<br><br>For additional demographic details, see the excerpt below from the manuscript's Supplemental Information:<br><br>Experiment 1:<br>After exclusions, a total of 720 participants were included in the final sample (Nmale = 360; Nfemale = 354, with 6 participants selecting “None of the above / Prefer not to identify”). Median age range in the final sample was 35-39 years of age (Q2 = 25-29, Q3 = 45-49, min = 18-19, max = 70-74). Median level of completed education was a bachelor’s degree (Q2 = “Some college but no degree”, Q3 = “Bachelor’s degree”, min = “Less than high school”, max = “Doctoral degree”). The majority of our participants identified as White (80%), followed by African American/Black (6.80%), Asian (6.25%), Multiracial (4.31%), Indigenous/Native American (0.55%), and 2.08% choosing not to identify.<br><br>Experiment 2:<br>After exclusions, a total of 320 participants were included in the final sample (Nmale = 201; Nfemale = 113, with 6 participants selecting “None of the above / Prefer not to identify”). Median age range in the final sample was 35-39 years of age (Q2 = 25-29, Q3 = 45-49, min = 18-19, max = 70-74). Median level of completed education was a bachelor’s degree (Q2 = “Some college but no degree”, Q3 = “Bachelor’s degree”, min = “Less than high school”, max = “Doctoral degree”). The majority of our participants identified as White (74.4%), followed by African American/Black (12.50%), Asian (6.25%), Multiracial (2.50%), Indigenous/Native American (2.19%), and 2.19% choosing not to identify.<br><br>Experiment 3:<br>After exclusions, a total of 80 participants were included in the final sample (Nmale = 17; Nfemale = 63). Median age range in the final sample was 30-34 years of age (Q2 = 20-24, Q3 = 40-44, min = 18-19, max = 60-64). Median level of completed education was a bachelor’s degree (Q2 = “High school degree or equivalent”, Q3 = “Bachelor’s degree”, min = “Less than high school”, max = “Doctoral degree”). The majority of our participants identified as White (72.5%), followed by African American/Black (13.75%), Multiracial (6.25%), Asian (2.5%), and 5% choosing not to identify.<br><br>Experiment 4:<br>After exclusions, a total of 160 participants were included in the final sample (Nmale = 76; Nfemale = 80, with 4 participants selecting “None of the above / Prefer not to identify”). Eighty participants were included in each condition: Emotion or Proofread. Median age range in the final sample was 35-39 years of age (Q2 = 25-29, Q3 = 45-50, min = 18-19, max = 70-74). Median level of completed education was a bachelor’s degree (Q2 = “Some college but no degree”, Q3 = “Bachelor’s degree”, min = “Less than high school”, max = “Doctoral degree”). The majority of our participants identified as White (81.88%), followed by African American/Black (8.75%), Multiracial (2.5%), Asian (1.88%), and 5% choosing not to identify.
Sampling strategy	This experiment used a convenience sample, as is standard practice for online cognitive psychological experiments. An advertisement was published to the AMT marketplace or Prolific. Participants were only allowed to participate once.<br><br>Due to the novelty and exploratory nature of our data collection, sufficient sample size was not determined a priori. Sufficient sample size was confirmed via replication of our main findings using several different stories, as reported in the main manuscript.
Data collection	Data were collected online using Psiturk, an open-access, publicly available, online experiment software. Participants performed this experiment from a web browser from their own desktop or laptop (mobile devices were not permitted). The experimenter was not present, nor able to watch/interact with the participants while they performed the experiment. Participants were randomly assigned to an experimental condition upon choosing to participate (automatically via Psiturk) and were not informed of the experimental manipulation until the end of the experiment. The experiment was synced with an SQL database hosted on a private server, which recorded each participant's data immediately after completion of the experiment.
Timing	This study consisted of four experiments, with multiple versions. Timings for each experiment and their variants are described below:<br><br>Experiment 1:<br>1012 participants took part in Experiment 1 and were recruited via Amazon Mechanical Turk (versions: Carver, Carver-Rewrite, July) or Prolific (version: Carver-Replication). MTurk data were collected over the span of June 2019 – March 2020. Prolific data were collected during September 2020.<br><br>Experiment 2:<br>769 participants took part in Experiment 2 and were recruited via Amazon Mechanical Turk. Data were collected during July 2020.<br><br>Experiment 3:<br>101 participants took part in Experiment 3 and were recruited via Prolific. Data were collected during January 2022.

	Experiment 4:<br>224 participants took part in Experiment 4 and were recruited via Prolific. Data were collected during January 2022.
Data exclusions	In line with recently published recommendations for online testing, we included a variety of data quality assurance indicators and excluded participants who did not meet these criteria (Chmielewski & Kucker, 2020). These were defined a priori. 292 participants were excluded in Experiment 1, 449 participants in Experiment 2, 21 participants in Experiment 3, and 84 participants in Experiment 4. Details regarding our exclusion criteria for each experiment can be found in the Supplemental Information in the "Participants and Exclusion Criteria" sections.
Non-participation	The experiment had an overall response rate of 81%; as 81% of participants who completed the consent form also completed the full 45-minute study. 61% of these participants were included in the final sample. For details regarding our exclusion criteria, see Supplementary Information: Supplementary Methods.
Randomization	Upon beginning the experiment, participants were randomly assigned to an experimental condition with equal probability. This was done automatically via Psiturk, the online experiment building platform used here.

Reporting for specific materials, systems and methods

We require information from authors about some types of materials, experimental systems and methods used in many studies. Here, indicate whether each material, system or method listed is relevant to your study. If you are not sure if a list item applies to your research, read the appropriate section before selecting a response.

Materials & experimental systems

n/a	Involved in the study
☒	☐ Antibodies
☒	☐ Eukaryotic cell lines
☒	☐ Palaeontology and archaeology
☒	☐ Animals and other organisms
☒	☐ Clinical data
☒	☐ Dual use research of concern

Methods

n/a	Involved in the study
☒	☐ ChIP-seq
☒	☐ Flow cytometry
☒	☐ MRI-based neuroimaging
